# Sfaira accelerates data and model reuse in single cell genomics

**DOI:** 10.1186/s13059-021-02452-6

**Published:** 2021-08-25

**Authors:** David S. Fischer, Leander Dony, Martin König, Abdul Moeed, Luke Zappia, Lukas Heumos, Sophie Tritschler, Olle Holmberg, Hananeh Aliee, Fabian J. Theis

**Affiliations:** 1 Institute of Computational Biology, Helmholtz Zentrum München, 85764 Neuherberg, Germany; 2 TUM School of Life Sciences Weihenstephan, Technical University of Munich, 85354 Freising, Germany; 3 Department of Translational Psychiatry, Max Planck Institute of Psychiatry, and International Max Planck Research School for Translational Psychiatry (IMPRS-TP), 80804 Munich, Germany; 4 Department of Mathematics, Technical University of Munich, 85748 Garching bei München, Germany; 5 Institute of Lung Biology and Disease and Comprehensive Pneumology Center, Helmholtz Zentrum München, Member of the German Center for Lung Research (DZL), Munich, Germany

**Keywords:** Single-cell genomics, Data zoo, Model zoo

## Abstract

Single-cell RNA-seq datasets are often first analyzed independently without harnessing model fits from previous studies, and are then contextualized with public data sets, requiring time-consuming data wrangling. We address these issues with sfaira, a single-cell data zoo for public data sets paired with a model zoo for executable pre-trained models. The data zoo is designed to facilitate contribution of data sets using ontologies for metadata. We propose an adaption of cross-entropy loss for cell type classification tailored to datasets annotated at different levels of coarseness. We demonstrate the utility of sfaira by training models across anatomic data partitions on 8 million cells.

**Supplementary Information:**

The online version contains supplementary material available at 10.1186/s13059-021-02452-6.

## Background

Many single-cell data sets are currently published in various databases in different formats, such as custom formats on GEO, manuscript supplements with tables of cell type annotations, or streamlined formats on Human Cell Atlas servers. Similarly, many parametric models for data integration, cell type annotation, and other tasks are published with their own user interface. The lack of streamlined data and model access inhibits data and model re-use and makes comparative analyses and benchmarks work-intensive. We identify two core issues with the current state of data and model re-use in single-cell genomics. Firstly, in smaller data sets, rare cell states can often only be properly analyzed after integration with larger reference atlas data sets. This integration is time-intensive and requires a prior analysis of the reference data set. The effectiveness of this approach depends on the reference atlas chosen. With a growing number of available reference data sets [1, 2], the choice of integration method and reference data set become increasingly hard to explore for analysts [3]. Secondly, data processing and cell type annotation are repeated elements of these pipelines that are time-intensive for analysts because of the complexity of the pipelines used^4^. Both computing an embedding and clustering require basic preprocessing, such as scaling, log-transformation, and highly variable feature selection. This data processing, also called feature engineering, is typically necessary both for basic embeddings such as t-SNE or uniform manifold approximation and projection [4] (UMAP) but also for embeddings from autoencoders such as DCA [5] and scVI [6]. Moreover, cell type annotation requires a high level of domain expertise as annotation resolution depends on the quality of the data and project requirements and because cell type ontologies are currently under development and therefore may change over the time scale of a typical analysis project.

We argue that zoos of pre-trained models can alleviate these problems by replacing processing steps that are usually manually tuned by analysts with standardized parametric models that correspond to entire processing pipelines. First, similar models can be trained on different data sets or collections, allowing analysts to navigate different reference data sets easily. Second, a zoo eases model sharing through a unified front-end. The idea of model sharing has been successfully applied in other fields including natural language processing and computer vision and in geomics with kipoi [7] for sequence-based models. Here, we introduce sfaira, a versatile repository that serves pre-trained scRNA-seq models. To train these models across tissues and organisms, we coupled the zoo with a data repository that includes data sets from multiple data providers with unified annotations. This data and model zoo permits streamlined access to data sets and pre-trained models. The presented sfaira framework defines a common nomenclature that covers feature spaces, data sets, and cell type ontologies. We leverage this data zoo to train models in an automated fashion across large numbers of tissues in two species and propose a mechanism that automatically accounts for different cell type annotation resolutions in cell type classifier models. Current model zoos are model class centric, thereby impeding side-by-side usage of different models, such as different autoencoder topologies. The sfaira model zoo is designed to be model agnostic and to simply be as a unified front-end for serving and receiving models, thereby enabling transfer of models from developers to users.

In addition to these practical advantages of a data and model zoo, we also address the issue of interpretability and generalizability of models. We provide a size factor-normalized, but otherwise non-processed feature space, for models, so that all genes can contribute to embeddings and classification and the contribution of all genes can be dissected without the issue of removing low variance features. We show that this approach allows us to relate the dimensions of the latent space to all genes. We also present models that have been fitted without covariates, such as organ or experimental assay, on extremely diverse data sets. We argue that such models require higher abstraction on the gene space compared to models that use covariates to remove variation between data sets. To our knowledge, this is the first instance in which such models could successfully be trained. Altogether, we expect sfaira to provide the important service of model reuse and broad model profiling across a diverse range of unified data sets.

## Results

### Sfaira provides data sets, models, annotation, and model parameters within a unified framework

**Fig. 1:**
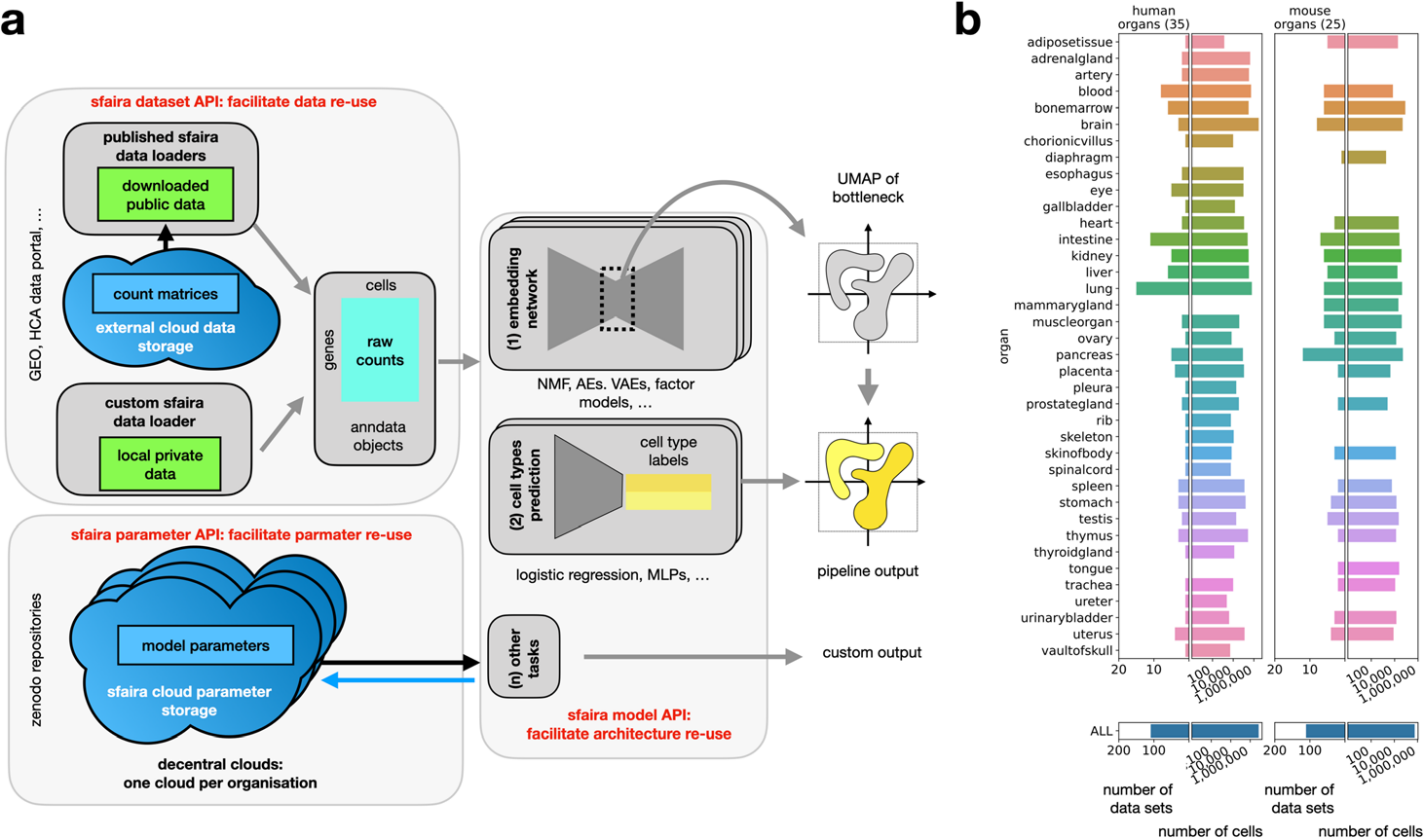
Sfaira is a data and model zoo that automates common steps in exploratory single-cell RNA-seq analysis. **a** Overview workflow of sfaira data and model API. Data set files are currently stored in cloud databases that can be interfaced with the sfaira data API to give streamlined AnnData objects that can be used for analysis or model fitting. The sfaira model API can consume these data sets to produce automatised analyses by querying parameter estimates from pre-trained models stored on cloud servers via the sfaira parameter API. Example analysis steps that are automated are embedding computation and cell type annotation. **b** Summary of the current state of sfaira data zoo for mouse and human single-cell RNA-seq samples, representing 220 data sets and 8.0 million cells in two organisms

Sfaira provides data, model, and parameter estimate access as a data and model zoo (Fig. 1a). Firstly, the data zoo contains dataset-specific loader classes to query data from the actual diverse data providers, which mirror data reading scripts and make these scripts sharable and reproducible. This data zoo is scalable because data loaders can be easily shared. Currently, as of May 2021, sfaira encompasses 41 studies with 220 data sets and 8.0 million cells (Fig. 1b). This data loader implementation allows streamlined querying of data sets based on meta features, such as organism or tissue sampled and experimental protocol. We enforced cell ontology [8] labels in this data universe to make cell type annotation relatable between data sets. Beyond the cell ontology, we also enforced ontologies for disease [9], anatomy [10], cell line [11], experimental method [12], and developmental stage [13, 14]. Importantly, such ontologies for meta data allow relational reasoning in the database which allows meaningful and intuitive subsetting in queries such as for all T cells, or for all samples from any lung tissue. The gene space is explicitly coupled to a genome assembly to allow controlled feature space mapping.

Secondly, the model zoo part of sfaira consists of a unified user interface and model implementations, not requiring the user to understand technical differences between models such as supervised cell type prediction models, matrix factorizations, and variational autoencoders. It is often desirable to use pre-trained models during analysis. For this purpose, we couple the pre-implemented models to parameter estimates stored locally or in a cloud database. These parameterizations can easily be queried from within Python workflows and allow streamlined execution of previously published models. The parameter query depends on a global model and parameter versioning system that we introduce with sfaira.

### A scalable data zoo for fast and comprehensive data access across numerous repositories

An important technical challenge faced by a data zoo is the interaction with large, heterogeneous data set collections that do not fit into memory. We address data loading by using streamlined data-set-specific loader classes that contain data-loading scripts. These classes can be written, maintained, and used in the context of the complex functionalities of parent classes, as well as shared through a single public code base. Moreover, we extend these data-set-specific loader classes to data collection loaders that serve streamlined data sets. Importantly, sfaira only requires a constant amount of code to load data sets, independent of the set of selected data sets. We also introduce lazy-loaded representations of data sets that allow users to subset large data set collections before loading desired subsets into memory. Here, we provide functionalities to write data sets with a streamlined feature space and metadata either into h5ad-based backed AnnData objects [15] or distributed data set collections that can be interfaced by distributed computing frameworks, such as dask (https://dask.org/). Last, we also aid data zoo exploration through a web front end that contains a searchable summary of all data sets in the data zoo database (*Availability of data and materials*).

### Scalable access to data with unified annotations allows for queries of gene and data statistics

**Fig. 2:**
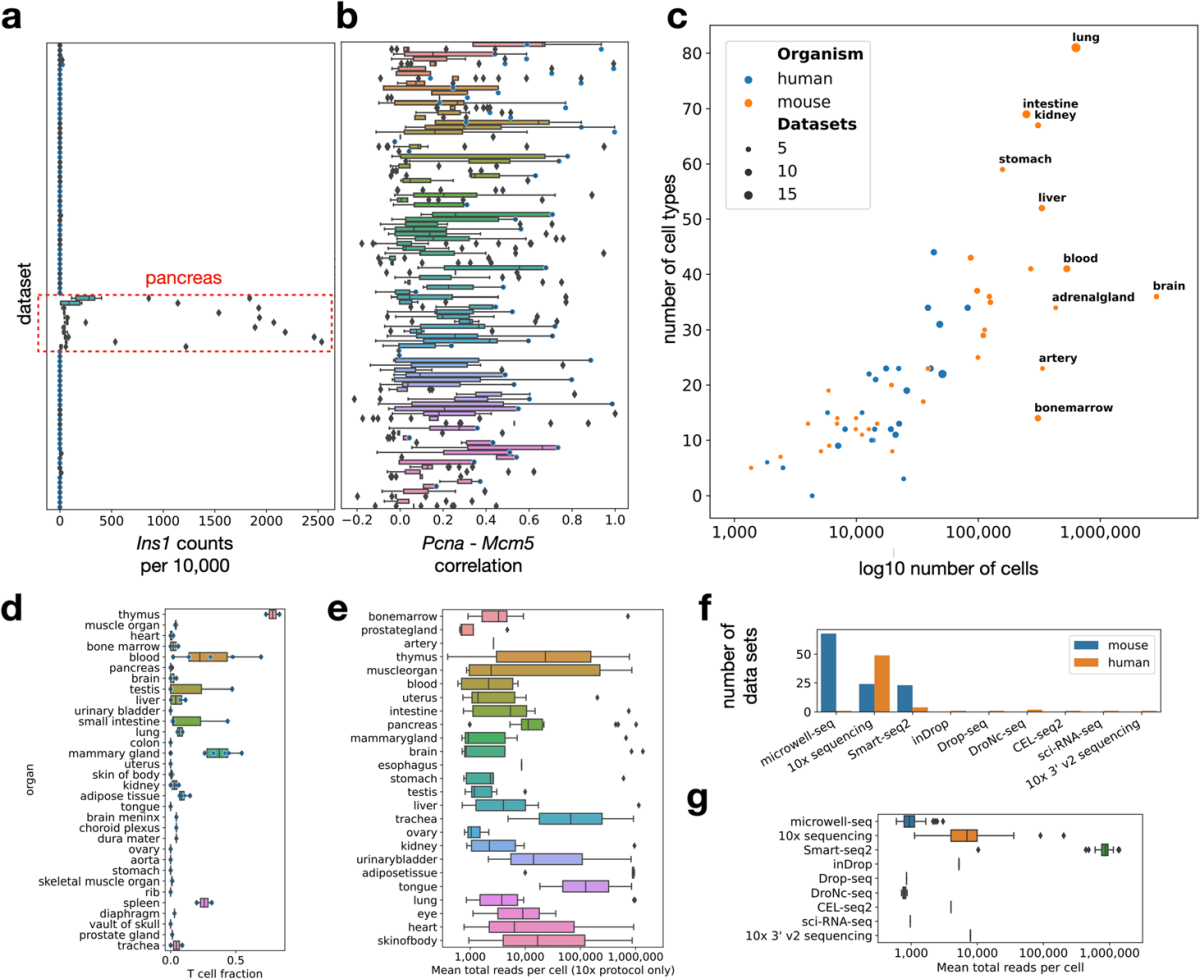
The sfaira data zoo contextualizes data statistics. **a** Characterizing gene expression distributions across organs. The expression range of the example gene insulin across mouse data sets shows specific expression in pancreas. Mean normalized expression of *Ins1* by organ and cell type. **b** Pearson correlation coefficient between the two cell-cycle-associated genes *Pcna* and *Mcm5* across data sets. Shown is a boxplot of the distribution over the correlation coefficients for each data set computed per organ and cell type. **c** Data sets vary strongly in complexity. Shown is the number of cells versus the number of cell types in the data zoo by organ for both mouse and human. **d** Sfaira allows querying of cell type fractions in tissue across organisms. Shown is the fraction of T cells per mouse dataset, ordered by organ. **e** Mean total counts per cell in mouse and human organs for 10x protocol data sets only. **f** Number of data sets per experimental protocol. **g** Mean number of counts (unique molecular identifiers if available, otherwise reads) per data set by experimental protocol

Streamlined access to unified, large, cross-study data sets as provided by sfaira allows for easy data statistics queries that can be helpful for putting observations in the context of other data sets. A common query in this context is gene based. For example, it is often useful to have a reference range for the expression activity of a gene observed with scRNA-seq. This can be done with sfaira via a straight-forward query, as showcased for *Ins1* scaled expression across organs and cell types in mice (Fig. 2a). Note that cell type-wise summary metrics are often much more useful for such workflows than cross-data set averages, which are skewed toward frequent cell types and are more useful than extrema, such as maximum expression, which are heavily influenced by the variance of the expression distribution. This analysis establishes an active range between 0 and 2500 counts per 10,000 unique molecular identifiers as an active range for *Ins1* expression in mice, with all expressing cell types located in pancreas data sets. Next, we consider gene-gene dependencies. Often, one is interested in the correlation of expression between genes to establish regulatory relationships. As an example, we investigated the correlation of two cell-cycle-associated genes, *Mcm5* and *Pcna* (Fig. 2b), which provides a range for their correlation and an estimate of how often these genes are correlated across tissues.

A second group of such queries is based on subsetting operations across cells based on cell and dataset metadata. Such queries depend on a homogenous annotation of metadata across data sets. Sfaira enforces this type of annotation by requiring meta data to follow ontologies. In sfaira, we also implemented relational reasoning of metadata items based on ontologies which is often necessary to achieve meaningful subsets. We showcase a few example data zoo summary statistics that exploit metadata-based queries (Fig. 2c–g). We generated complexity plots of the total number of cells versus the number of most fine grained cell type labels per organ to give guidelines for prioritization of organs for further cell type discovery (Fig. 2c). In a cell type-centric scenario, we queried the fraction of T cells across organs (Fig. 2d), a query that can be used to characterize specific cell types across organs and datasets. Last, we queried a summary of total reads per cell summary statistics and protocol summary statistics (Fig. 2e–g).

### Sfaira enables automated single-cell data analysis

**Fig. 3:**
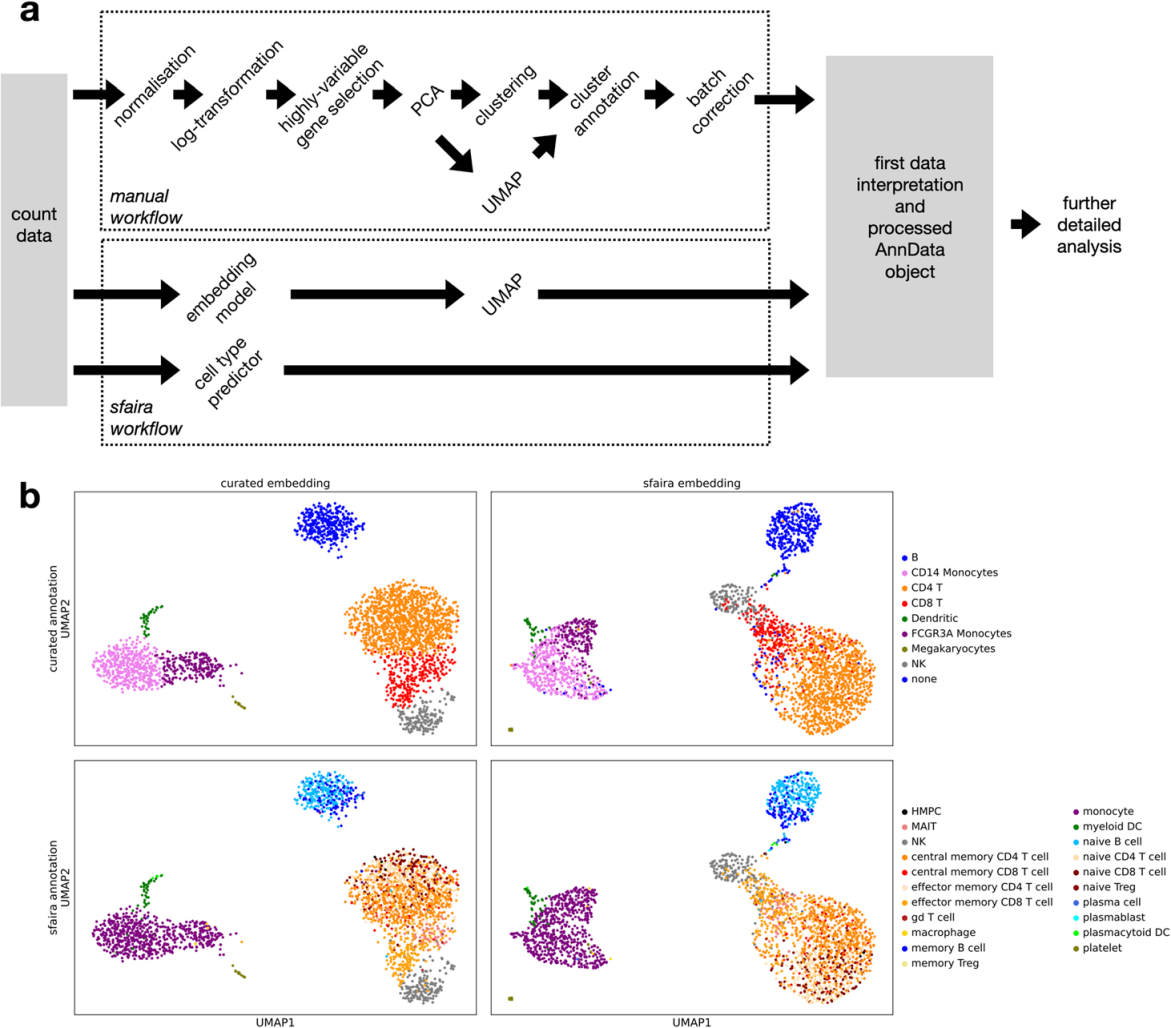
Sfaira automatizes exploratory analysis of single-cell data. **a** Manual single-cell data analysis pipeline and automated sfaira pipeline. **b** Comparison of manual feature engineering workflow with automated embedding and cell type annotation from sfaira on a human PBMC data set. Shown are UMAPs based on a PCA of an engineered feature space and of the out-of-the-box latent space from a linear sfaira embedding model. Superimposed are cell types previously annotated for this data set and sfaira cell type predictions

A core advantage of end-to-end parametric approaches is that they can alleviate the need for feature engineering. This has been a key advancement in image-based deep learning for example [16]. In single-cell analyses, feature engineering describes the early analysis steps starting from count matrices, including normalization, log-transformation, gene filtering, selecting components from principal component analysis (PCA), and batch correction [4, 17] (Fig. 3a). These steps are usually necessary to obtain useful embeddings and clusterings^4^ but are a bottleneck in analysis workflows. Pre-trained embedding models can be used to generate latent spaces that can be used for downstream tasks without prior feature engineering. As an example case, we processed human peripheral blood mononuclear cells (PBMC) data in a standard preprocessing workflow [4, 15] and compared this to a UMAP of a linear model embedding. Both the manual and the learned embedding separated annotated cell types into distinct clusters, which demonstrates that both captured the biological heterogeneity of the system (Fig. 3b). We performed four additional such zero-shot analysis examples on data sets not used for training or testing of the models presented (Additional file 1: Fig. S1) [18–21]. One could judge the learned embedding also based on the reconstruction error of its encoder-decoder model: Here, the linear model achieved a mean negative log likelihood in reconstruction of 0.16. These quantitative metrics on embedding models are necessary to compare multiple models. Second, we used automated cell type annotation to label cells to explore whether we could seed data interpretation with a first proposal for cell types. Cell type predictions from a multi-layer perceptron model trained on different data sets identified similar cell types to the labels from the curated annotation (Fig. 3b). Note that with further additions to the data zoo and improved classifier models trained on these large data sets, these coarse initial annotations will become increasingly fine-grained. This example shows that the combination of pre-trained embedding and cell type classifier models can be used to perform an automated initial analysis of single-cell data, which can then be extended by further in depth analysis according to the scenario. Below, we discuss pre-training details of such cell type classifiers and embedding models that allow these workflows on a large scale.

### Sfaira versions decentralized parametric models to allow reproducible model sharing and application to private data

Sfaira implements two model classes: (i) gene expression reconstruction models that learn a latent representation that can be used for visualization, and (ii) supervised models that predict cell type labels (Fig. 1a). The model classes are defined by their input and output. Sfaira’s architecture can also integrate other model classes that serve additional purposes. Models are characterized by an input feature space, an output space, and model architecture hyperparameters. Importantly, we make input feature space standardization easy by coupling input gene sets to genome assemblies and functional annotation of gene sets. One can for example define an input feature space as the protein coding genes in GRCh38 version 102 (Fig. 1a). The label space of cell type classifiers is a set of cell types in the cell ontology [8]. This label space is a set of leaf nodes of a subgraph of the full ontology graph and thus makes hierarchical labels defined in the ontology available to the cost function. We broadly categorize model topologies according to popular approaches: matrix factorizations, autoencoders, and variational autoencoders for reconstruction models and logistic regression and densely connected neural networks for cell type classification.

We provide an infrastructure for third party organizations to maintain their own public and private repositories of model weights (model zoos) on servers or in local directories. These parameter set versions are identified by the organization that performed model training as well as the training data and optimization hyperparameters that this organization used to train this model. Often, this would result in organizations providing an initial estimate that becomes incrementally updated as new data becomes available or when improved estimates become available in an ongoing grid search across optimization hyper-parameters. Sfaira allows end-users to easily switch between different model types from different model providers, accelerating and democratizing model distribution and access. This reduction in the effort required to quickly implement and compare models will improve decisions on pre-trained model usage. In addition, the decentralized storage of model weights allows this model zoo to quickly react to new developments in the community.

### Generalized cell type prediction within an ontology adjusts for annotation coarseness

**Fig. 4:**
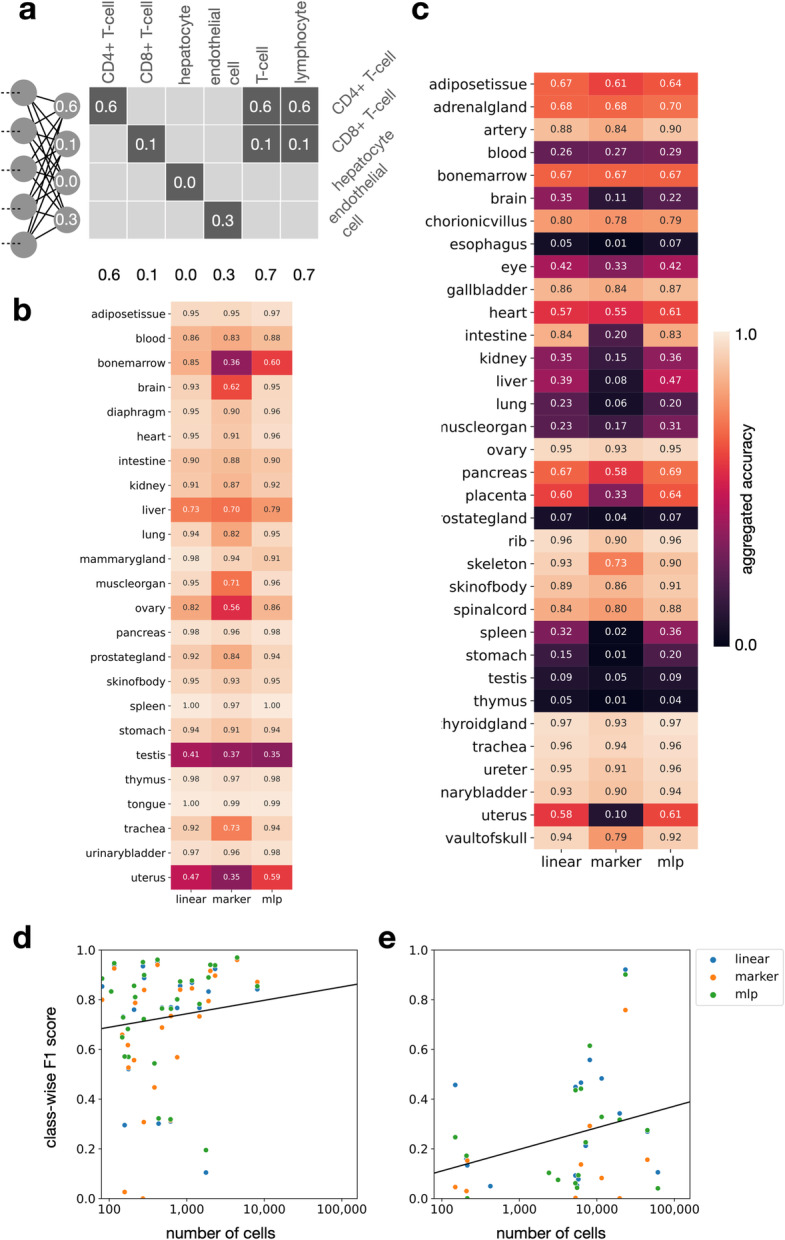
Sfaira allows fitting of cell type classifiers for data sets with different levels of annotation granularity by using cell type ontologies. **a** Aggregated accuracy and cross-entropy allow for fitting cell type classification models on data sets with heterogeneous annotation coarsity using cell type relations from ontologies (see the “Methods” section). The y axis contains leaf nodes of a cell type ontology, which can be combined linearly to yield the predicted probability mass of any other node in the ontology graph (*x*-axis). **b**, **c** Accuracy of cell type classifiers on mouse (**b**) and human (**c**) organs on entirely held-out test-data sets. Linear: Linear classifier (logistic regression), marker: Marker gene-based classifier, MLP: multilayer dense neural network. **d**, **e** Class-wise prediction accuracy correlates with the number of cells in class. Shown are cell type class-wise F1 scores by the number of cell types in that class of cell type classifiers by model on lung data from mice (**d**) and humans (**e**)

A core difficulty for deploying predictive models for cell type labels based on single-cell RNA-seq is that cell type labels can change as part of ongoing cell atlas efforts [22]. We address this issue by defining models on specific versions of the cell ontology [8] and allow extensions of this ontology to keep up with non curated developments. A second challenge is that cell type annotation from previous studies is often presented at different resolutions. One study might report “leukocytes” in a given tissue while a different study differentiates between “T cells” and “macrophages.” A scalable training framework for cell type classifiers needs to be able to make use of both levels of granularity, as manual re-annotation is time-consuming and may not always achieve the required resolution, depending on data quality. This notion of coarseness relates to the directed acyclic graphs that are typically employed in cell type ontologies. Accordingly, we propose the usage of a variant of cross-entropy loss and an accuracy metric that can dynamically assign observations to single labels or to sets of labels during training and testing (aggregated cross-entropy, Fig. 4a, see the “Methods” section). Using this approach, we were able to pool cell type annotations from more than 149 public data sets and train predictive models for 24 mouse tissues and 34 human tissues across 6.6 million cells at once.

It has recently been proposed that cell type prediction can often be performed with linear models [23]. We trained three types of models: logistic regression models, multi-layer densely connected feed forward neural networks (multi-layer perceptrons), and a new marker gene-centric linear model (Methods). The newly proposed gene-centric model operates in a learned marker gene space in which each gene is first transformed into a binary on-off state with a sigmoid mapping. Such models are not only easy to interpret, as marker genes contribute equally to the prediction, but they also allow integration of prior knowledge on marker genes via priors for the parameters of the marker state embedding layer. All models performed well as expected based on previous findings [24] on selected organs (Fig. 4b,c), with a median accuracy of 0.64 in human samples and 0.93 in mouse samples. We did not find performance advantages of the marker model. Our data zoo facilitates training and deployment of these models in a streamlined fashion, thus making cell type predictors easily accessible for all sampled organs and organisms. Using the data zoo, we can easily relate classifier performance to class frequencies (Fig. 4d,e) and can consider individual classes in more detail (Additional file 1: Fig. S2).

### Sfaira serves embeddings from different models

Embedding models compress data to a low-dimensional representation which is necessary for many downstream analyses. Members of this model class that have been used frequently in the past for representation learning on single-cell RNA-seq are PCA, non-negative matrix factorization [25, 26], autoencoders [5], and variational autoencoders [6]. Embedding models have been successfully used in the context of transfer-learning [25, 26], a process during which public data are leveraged to improve learned representations. Still, workflows that use such encoder-decoder models in unsupervised scRNA-seq data analysis usually rely on refitting the model on each new data set for two core reasons: First, useful pre-trained models are difficult to identify in the literature. Second, unsuccessful transfer training of pre-trained models may result in relevant variation of the data set not being resolved, such as new cell states. Sfaira serves embedding models in a structured fashion to users and exposes a large data library for pre-training, thus reducing the probability that components of variation which are relevant to the test task were not seen during training. Here, we benchmark such models on a large data collection to show that we can indeed address these two issues.

**Fig. 5:**
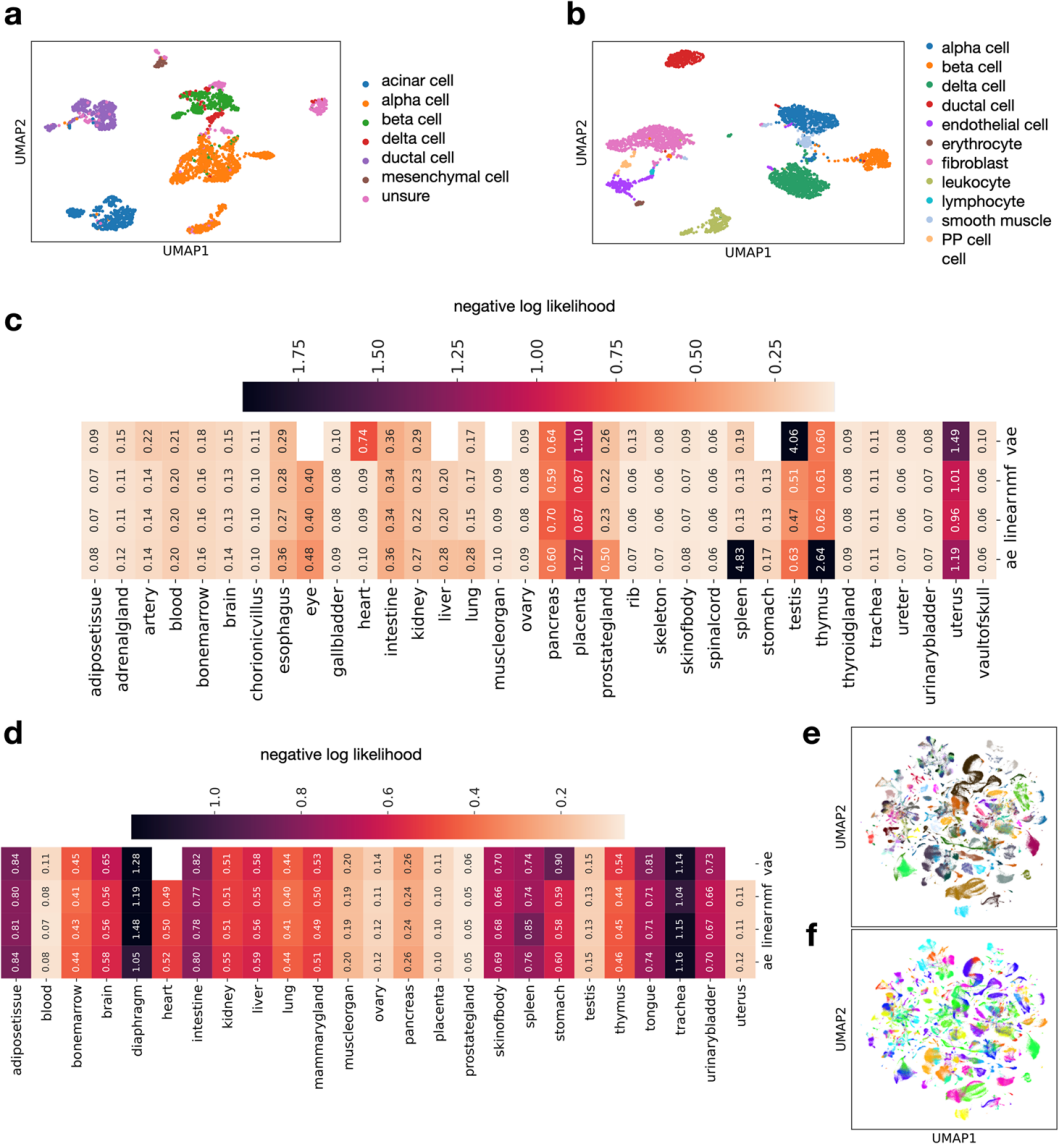
Sfaira allows streamlined embedding models training across tissues and on whole atlases. **a**, **b** Pre-trained embedding models can perform meaningful reconstruction of cells in held out data sets. UMAP based on latent space of the best embedding model data for pancreas data from humans (**a**) and mice (**b**). The superimposed colors correspond to the original, non-streamlined, cell type annotation. **c**, **d** Reconstruction performance comparison of different embedding models across organs and organisms. The negative binomial likelihood is used as a reconstruction performance metric on reconstructed test data of held-out test-data sets from PCA, linear, non-negative matrix factorization (nmf), autoencoder (ae), and variational autoencoder (vae) models on human (**c**) and mouse (**d**) organs. **e**, **f** Sfaira allows for training of embedding models using very large data sets. UMAP of the latent space of an embedding model trained on all mouse data in the sfaira data zoo with the data set (**e**) and cell type (**f**) superimposed

Where possible, we defined hold-one-data-set-out test splits across organs to reflect the ability of these models to capture variance in settings with previously not seen confounding effects. Example embeddings for human and mouse lung data sets (Fig. 5a,b) show that cell types are separated. We then compared reconstruction errors in cross-validation splits across commonly used model classes across 35 human tissues and 25 mouse tissues, using four different classes of embedding models. We found that linear models perform similarly to non-linear models, with median best achieved negative log likelihood of linear models and organs for human samples of 0.13 and for mouse samples of 0.50 (Fig. 5c,d). Best achieved negative log likelihood for human blood models was also 0.08 for linear models, which is of similar magnitude to the reconstruction error found on the held-out PBMC data shown in the automated example analysis (Fig. 3b). These models perform better than baseline random projection models (“Methods” section, Additional file 1: Fig. S3). This finding shows that single-cell data can be reconstructed well by pre-trained linear models [23]. In sfaira, we improve embedding analyses in three aspects. By deploying pre-trained models that are already optimized for hyperparameters, we alleviate the need for grid searches or feature engineering. Second, we reduce the burden for model interpretation as previously annotated model components, such as bottleneck dimensions, can be easily leveraged for new analysis, thereby adding value to an analysis that goes beyond representation capabilities. Third, by enabling training on extremely diverse data sets, we pave the way for the usage of highly interpretable models that are more difficult to train. The embedding models shown here are examples of models that can be used in a model zoo but do not represent the full range of pre-trained models that could be used in the single-cell context [27].

### Regularizing models through organism-level data

Data integration is a trade-off between removing between-sample variance resulting from technical effects and conserving biologically meaningful variance [3]. Instead of removing between-sample variance in a data integration setting, we focus on embedding models that discover axes of variation which allow us to discern biological variation on a new data set (zero-shot learning [28]). Here, it is difficult to discern models that overfit all variation in data sets and models that capture only relevant axes of variation. This overfitting is an issue that can be addressed through regularization. Model regularization in embedding and cell type classifier neural networks is often performed via L1 or L2 constraints on model parameters, via drop-out mechanisms [5], and via dimension bottlenecks in latent representations. While effective in the prevention of overfitting, these regularization methods cannot be easily used to derive interpretable models. Instead, they dynamically limit the degrees of freedom of generously over-parameterized models.

In principle, models can also be regularized through extremely diverse training data, thus making it hard for the model to overfit the entire training domain and forcing the model to learn strong abstractions. Importantly, this data-driven approach stands in contrast to the usage of variance explaining covariates in conditional embedding models: An embedding model with a high degree of abstraction should be able to learn abstract representations of gene expression configurations across conditions, similar to how image-trained convolutional networks learn representations of images from different objects or sources without having access to categorical descriptors of these conditions. Conditional embedding models are often used in data integration studies, in which domain differences are usually removed by a projection mechanism [29]. Sfaira provides structured data libraries built for providing models with extremely large training data sets. Indeed, we could converge embedding models on such large data zoos of scRNA-seq data of whole organisms across datasets from many studies (Fig. 5e,f). In summary, sfaira is well positioned to enable model regularization through, extremely diverse training data sets, with the aim of extending reference data usage from projection-based data integration to more abstract pre-trained embeddings.

### Embedding model interpretation through gradient maps from bottleneck to input features

**Fig. 6:**
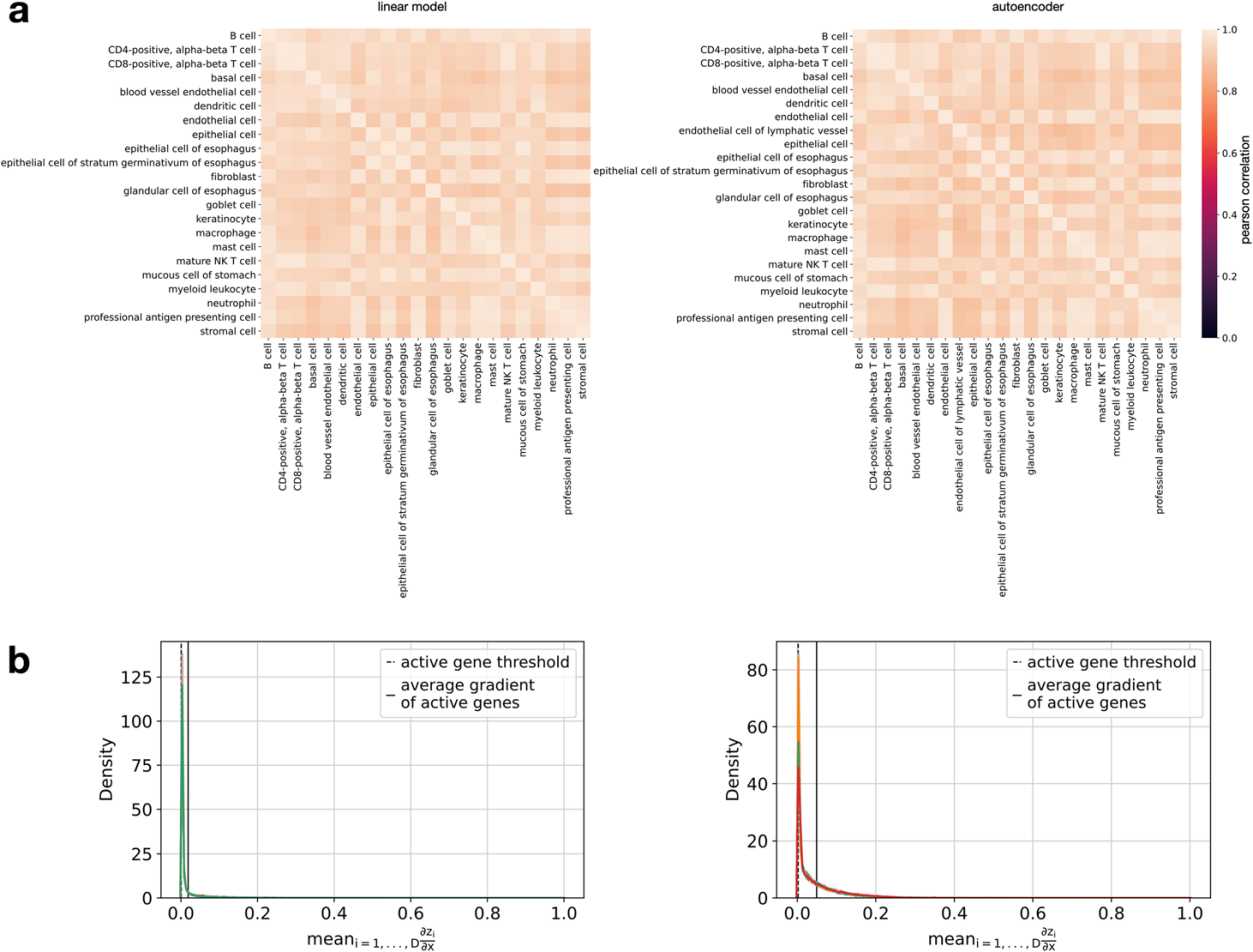
Toward the interpretability of model embedding. Saliency-based interpretation and data regularization of non-conditional embedding models: linear and autoencoder embedding models for human esophagus (**a**, **b**). **a** Correlation of cell-type wise aggregated gradients of embedding with respect to input features. **b** Distribution of feature-wise wise aggregated gradients of embedding with respect to input features by cell type (color)

Many embedding models that are used in single-cell RNA-seq have been based on PCA. PCA is desirable as an embedding in terms of interpretability, because it allows for a direct interpretation of latent dimensions as orthogonal linear combinations of the input features (loadings). Gradient maps from the bottleneck activations to input features allow locally similar interpretation mechanisms in non-linear embeddings of encoder-decoder networks. Such gradient maps carry the promise of correlating bottleneck dimensions to molecular pathways or similar complex regulatory elements that present a higher-level view of gene regulatory networks. We found that cell-type-wise gradient maps of the embedding space with respect to the feature space revealed cellular ontology relationships in two sample data sets (Fig. 6a, Additional file 1: Fig. S4a) by grouping similar cell types together within a hierarchical clustering of the gradient correlation matrix. Moreover, we found that linear models and autoencoders are similar in the size of feature sets considered important by these gradient-based mechanisms for each cell type (Fig. 6a, Additional file 1: Fig. S4a) and also have a similarly shaped marginal distribution of normalized gradients (Fig. 6b, Additional file 1: Fig. S4b). Models trained only with small data sets may collapse to only use small subsets of the gene space and represent cells based on feature correlations in this feature space. As data sets grow, more complex representations have to be learned, and any collapse of models on sub-feature spaces can be diagnosed with gradient-based approaches.

## Discussion

We introduced sfaira, a data and model zoo which accelerates and standardizes data exploration for scRNA-seq data sets. The automated exploratory analysis aspect of sfaira workflows smoothly integrates with scenario-specific scanpy [15] workflows and scales data exploration by reducing the number of manual steps performed by analysts. Sfaira accelerates parallelized model training across organs, model benchmarking, and comparative integrative data analysis through a streamlined data access backend while improving deployment and access to pre-trained parametric models. The mechanism introduced here to accumulate large reference databases and to fit models on extremely diverse data set collections, provides a gateway to regularization through data and to mechanistic models. In contrast to query-to-reference analysis, the models presented here can be leveraged for unconstrained data exploration [29, 30]. Lastly, our framework is open to the contribution of single-cell centric models that do not primarily serve the purpose of single-cell RNA-seq embedding or cell type prediction. Other use cases may include embedding models across multi-modality joint feature spaces such as CITE-seq [31] or cell doublet prediction [31–33]. We used model deployment infrastructure from TensorFlow (https://www.tensorflow.org/) here, similar infrastructure is available from PyTorch (https://pytorch.org/), and both are very simple to maintain. One could also think about deploying executable models in Docker (https://www.docker.com/) images, as is currently done in kipoi for functional genomics data [7].

## Conclusion

Our effort to streamline the zoo of single-cell data is complementary to institutionalized efforts, such as the Human Cell Atlas. Our mixed data zoos can represent every data set in a publicly maintained, data-set-specific code base, and, at the same time, can leverage consistent data representations from data providers, while retaining a single interface. In a partnership with cellxgene [34], we built conversion code to translate sfaira dataset to cellxgene formatted datasets and conversely, thus allowing processed data storage on the cellxgene cloud servers and interfacing additional datasets provided by cellxgene. Moving beyond scRNA-seq, we will support different data modalities such as from splicing annotation, scATAC-seq [35], CITE-seq [31], and spatial molecular profiling in the near future. We expect sfaira to become a useful resource for automated data analysis, a comprehensive source of reference data sets, and to enable benchmarking of new methods. The models proposed for automated data analysis are likely to improve drastically with increasing availability of training data resulting in strong performance improvements in the near future as the data zoo grows.

## Methods

### Implementation

#### Data zoo

We represent data sets by individual data loader classes that inherit generic data-loading properties from a unique parent class. These data loader classes can be considered class versions of data-loading scripts that are otherwise often used in script code. These classes allow metadata queries through automatic metadata storage in a lazy mode, in which count matrices are not yet loaded into memory, thereby allowing the user to subset large instance lists of these classes interactively. Some entities serve streamlined processed data sets for which individual loading scripts are not necessary: In these cases, we interface these data zoos with a single class that can be instantiated for all data sets in this zoo. Sfaira maintains the universe of all contributed data loader classes; users then locally build libraries of a subset of these data sets, and the sfaira data api accesses all available data sets: This allows users to also only operate on a subset of the available processed data universe.

#### Model zoo

We provide a model code in the sfaira package; each model has its own model class that can be accessed through a streamlined interface, such as in *kipoi* [7].

#### Parameter storage

Parameter files of models defined in the sfaira model zoo are stored in public cloud servers, such as Zenodo, or locally for private models. These parameter files are versioned and can thus be reproducibly accessed.

### Model topologies

Sfaira is a model zoo that is set up to accommodate various models. Here, we describe the models that underlie the analysis results that are presented in this manuscript. Note that the models in sfaira will not be limited to this initial model population in the future.

#### Preprocessing layers

We prepended a common input data transform to all embeddings and cell type prediction models. The objective for using this transform is to reduce variability in the data so that models require lower complexity and fewer training steps to adjust their internal normalization of the data. We chose a transform that can be evaluated based on a data batch without being dependent on the batch. For arbitrary batch sizes, this requires the transformation of an individual observation (cell) to only depend only on the observation itself. We linearly scaled the data points *x* per cell *i* and gene *j* to 10,000 and log transformed this scaled vector.

$$ {x}_{ij}=\mathit{\log}\left(\frac{x_{ij}}{\sum_{n=0}^N{x}_{in}}\ast {10}^4\right) $$

The scaling is a basic attempt to reduce the variation caused by the number of UMIs observed per cell which depends on technical factors such as the library depth and stochasticity in mRNA capture during the sequencing experiment. The log transform is a basic attempt at reducing the strong heteroscedasticity of the data which is commonly observed to have a positive dependence of the variance on the mean of the gene observations.

We would like to highlight that unlike in standard single-cell RNA-seq data processing for PCA and downstream t-SNE or UMAP computation, this processing does not necessarily need to be carefully benchmarked as this processing is complemented by the innate ability of the first layers of the neural network to adjust to unwanted sources of variation. We chose to use a basic transform to speed up training only. In the limit of many data sets and sufficient training time, one could imagine entirely removing preprocessing from these networks.

#### Output and loss function of embedding models

We provide support for different model outputs and loss functions. These variations are encoded in the topology identifier. Multiple studies have found that autoencoder can learn embeddings of single-cell RNA-seq data with negative binomial reconstruction loss. A negative binomial reconstruction loss requires a mean *μ* and a dispersion parameter *φ* to be estimated. In the initial version of sfaira, we support output states tailored to the negative binomial distribution through an exponential inverse-linker function in the last layer. We distinguish an output that estimates a fixed dispersion per gene and an output that estimates one dispersion parameter per gene and cell. The negative log likelihood over *N* samples and *J* genes is ad follows:

1 $$ {\mathrm{ll}}_{\mathrm{NB}}\left(\mu, \upvarphi; x\right)=-{\sum}_{0\le n\le N}{\sum}_{0\le j\le J}\log \kern0.2em \Gamma \left({\upvarphi}_j+{x}_{nj}\right)+\log \kern0.2em \Gamma \left({x}_{nj}+1\right)+\log \kern0.2em \Gamma \left({\upvarphi}_j\right)-{x}_{nj}\ast \left(\log \left({\mu}_{nj}\right)+\log \left({\mu}_{nj}+{\upvarphi}_j\right)\right)-{\upvarphi}_j\ast \left(\log \left({\upvarphi}_j\right)+\log \left({\mu}_{nj}+{\upvarphi}_j\right)\right) $$

#### Output and loss function of cell type prediction models

The standard cell type prediction model included in sfaira operates under the assumption that a cell type prediction should output a probability distribution across previously known cell types. The loss typically used for evaluating fits of such probability mass distributions is the cross-entropy loss. We additionally allow for multiple output categories to be assigned to a single true set of labels, we call this aggregated cross-entropy loss and we use aggregated accuracy as an evaluation metric for this scenario. This aggregation is necessary if data sets differ in the coarseness of the cell type assignments. Often, one can map labels between both data sets as part of an ontology. Data set A may only annotate four tissue-specific cell types and “lymphocytes” whereas data set B differentiates those four types and further differentiates “T cells” and “B cells”. The cell universe of this tissue should, therefore, consist of the four tissue-specific cell types and T cells and B cells. Data set A can still be used to train supervised classifiers to predict cell types, but one must take care that the lymphocyte label is used properly. We propose to aggregate the predicted class probabilities across all labels assigned to lymphocytes in data set A so that any probability mass distribution for a lymphocyte observation in A across T-cells and B-cells is allowed. This allows the classifier to learn differences between T-cells and B-cells on data set B, while it can use A to improve its model of the difference between both lymphocytes and the remaining four cell types. Below, we compare the resulting aggregated cross-entropy loss cce_agg_to cross entropy for a binary (cce_binary_) and a multi-class (cce_multi − class_) prediction problem. The shown transformations labeled with (*) hold if *y* ⊂ {0, 1}, ie., if the labels lie on a binary support.

2 $$ {\mathrm{cce}}_{\mathrm{binary}}=-{\sum}_{0\le n\le N}{y}_n\ast \log \left({p}_n\right)+\left(1-{y}_n\right)\ast \log \left(1-{p}_n\right)\overset{\left(\ast \right)}{=}-{\sum}_{0\le n\le N}{\sum}_{k\subset {K}^{+}}\log \left({p}_{nk}\right) $$

3 $$ {\mathrm{cce}}_{\mathrm{multi}-\mathrm{class}}=-{\sum}_{0\le n\le N}{\sum}_{k\subset K}{y}_{nk}\ast \log \left({p}_{nk}\right)\kern0.2em \overset{\left(\ast \right)}{=}-{\sum}_{0\le n\le N}{\sum}_{k\subset {K}^{+}}\log \left({p}_{nk}\right)\overset{\left(|K|=2\right)}{=}{\mathrm{cce}}_{\mathrm{binary}} $$

4 $$ {\mathrm{cce}}_{\mathrm{agg}}\kern0.2em {=}^{\left(\ast \right)}-{\sum}_{0\le n\le N}\log \left({\sum}_{k\subset {K}^{+}}{p}_{nk}\right)\overset{\left(\forall n:{\sum}_{0\le k\le K}{y}_{nk}=1\right)}{=}{\mathrm{cce}}_{\mathrm{multi}-\mathrm{class}}, $$

where *K*^+^is the set of positive classes with *y*_*k*_ = 1and *K*^−^is the set of positive classes with *y*_*k*_ = 0 and *N*is the number of observations. In the above example with lymphocytes that are split into T cells and B cells, ∑_*k* ⊂ *K*_*y*_*k*_ = 2 for observations assigned as lymphocyte, as the label is *y*_*k*_ = 1 for both the T cell and B cell class which make up the set of lymphocytes *K*^+^. Similarly, the predicted probability mass for an observation *n* that is labeled lymphocyte is the sum of probability masses predicted for T cells and B cells ${\sum}_{k\subset {K}^{+}}{p}_{nk}$. In contrast, T cell is a leaf node label and its set of positive classes *K*^+^ only contains the label T cell. Here, the predicted probability mass for the label T cell is ${\sum}_{k\subset {K}^{+}}{p}_{nk}={p}_{nl}$ where *l* is the T cell class. The accuracy metric acc_agg_corresponding to cce_agg_is:

5 $$ {\mathrm{acc}}_{\mathrm{agg}}=-\frac{1}{N}{\sum}_{0\le n\le N}I\left[\left({\sum}_{k\subset {K}^{+}}{p}_{nk}\ast {y}_{nk}\right)\succ {\max}_{k\subset {K}^{-}}\left({p}_{nk}\right)\right], $$

where *I*[] is an indicator function, which assesses whether the aggregate probability mass predicted for a given cell type label is larger than the probability mass assigned to any leaf node of the ontology that is not a subclass of the class in question.

Alternatively, one could use sigmoid transforms of independent cell type predictions. This approach does not superimpose the prior knowledge that a cell can only be part of one class in a properly defined cell type ontology, and thus, we therefore do not support this setting.

#### Multilayer perceptron model

We used dense layer stacks (multilayer perceptron) to predict cell types from gene expression data. An example multilayer perceptron for cell type prediction used in this study was trained on all protein coding genes from either mouse or human, had one hidden layer of the size (128), was trained without L1 and L2 penalties on the parameters, and with a selu activation function.

#### Marker model

We defined a marker gene-dominated model to predict cell types from gene expression data. In this model, a sigmoid function based on a gene-specific linear embedding of the gene expression values models an expression threshold. After this gene-wise embedding, a fully connected layer pools information from all genes to the cell type prediction.

#### Autoencoders

Autoencoders with “dense” (fully-connected) layers and count noise distributions were proposed among others by Esralan et al. to learn embeddings of single-cell RNA-seq data [5]. The full architectures are documented in the code. An example autoencoder used in this study was trained on all protein coding genes from either mouse or human, had three hidden layers of the sizes (512, 64, 512), was trained without L1 and L2 penalties on the parameters and without input drop-out, was trained with batchnorm between dense layers, with selu activation function, and with a single trained dispersion parameter per gene in the output for the negative binomial reconstruction loss.

#### Variational autoencoders

Variational autoencoders (VAEs) with “dense” (fully-connected) layers on count noise data were proposed among others by Lopez et al. to learn embeddings of single-cell RNA-seq data [6]. Here, we impose a unit Gaussian prior on the latent space activations. The full architectures are documented in the code. An example variational autoencoder used in this study was trained on all protein coding genes from either mouse or human, had three hidden layers of the sizes (512, 64, 512), was trained without L1 and L2 penalties on the parameters and without input drop-out, was trained with batchnorm between dense layers, with selu activation function, and with a single trained dispersion parameter per gene in the output for the negative binomial reconstruction loss.

#### Random projection

We use random projection as a baseline embedding model, in order to put our reported model performance into context. For this we use the sklearn.random_projection.SparseRandomProjection() method from scikit-learn (v0.24.1). As with all other models, we fit the model on the training data and project the test data to reduced dimensions (64 in this case). We then reconstruct the original dimensionality of the data by multiplying the reduced data with the components of the fitted random projection model. For numerical reasons, we consider any negative values in the reconstruction as invalid values and convert them to a small positive number (1e−10). We do the same for any zero values in the reconstruction in order to allow computation of the losses. We then compute the mean squared error of the reconstruction as well as the negative log-likelihood of the negative-binomial distribution with a constant scale of 1.0.

### Data processing

#### Expression data

All data (human [30, 36–71] and mouse [72–75]) were downloaded in the least-processed expression matrix format provided by the authors of the data set. Their processing is documented in their respective data loaders within sfaira. The datasets used for example zero-shot analyses (Fig. 3b, Additional file 1: Fig. S1) were downloaded from scanpy [15] from and as cellxgene data collections [18–21], as described in the accompanying notebooks. We did not perform any processing other than that discussed for preprocessing layers discussed in the section “Model topologies.” As feature space we chose the protein coding genes from the Mus_musculus.GRCm38.102 genome assembly for mice and Homo_sapiens.GRCh38.102 for humans.

#### Cell type annotation data

Not all data sets used in this manuscript use the same cell type identified conventions. We mapped the cell type annotation from each data set to the cell ontology. We defined the label space of each cell type predictor model per anatomic location based on the most fine grained cell types observed in this dataset: If one considers the directed acyclic graph of the ontology, these label cell types are leaf nodes of a sug-graph that describes all cells observed in a given tissue and their ontological relationships. The loss and accuracy of coarser labels during testing and evaluation was evaluated using the aggregated cross-entropy and accuracy metrics described in the section “Output and loss function of cell type prediction models”.

#### Test data splits

Where available, entire data sets were held out to evaluate model performance test metrics. Some organs were only represented by a single data set in the data zoo. In these cases, we held out a random set of 20% of all cells as test data.

## Supplementary Information

**Additional file 1.** Supplementary Figures 1-4. Fig. S1: Zero-shot analysis example cases. Fig. S2: Characterization of the cell type classification task. Fig. S3: Random projection models as baseline models for cell embedding models. Fig. S4 Saliency-based interpretation of models trained on human kidney.

**Additional file 2.** Review history.

## Data Availability

The python package sfaira is available via GitHub [76] (https://github.com/theislab/sfaira) and available under a BSD-3-Clause license, the version used for this manuscript is also available from PyPi [77] (https://pypi.org/project/sfaira/0.3.0/). The model fits discussed are available from zenodo [78] (https://zenodo.org/record/4836517). An overview of the data zoo is provided here (https://theislab.github.io/sfaira-portal/). The notebooks containing the analysis results presented here are available at https://github.com/theislab/sfaira_benchmarks. Tutorials are available at https://github.com/theislab/sfaira_tutorials. Data sets [30, 36–75] were downloaded as described in the “Methods” section.
