# Supplementary material for: Sfaira accelerates data and model reuse in single cell genomics
Source: Genome Biol. 2021 Aug 25;22:248. doi: 10.1186/s13059-021-02452-6 (PMC8386039; doi:10.1186/s13059-021-02452-6)
Supplement: Supplementary file 1 — Additional file 1. Supplementary Figures 1-4. Fig. S1: Zero-shot analysis example cases. Fig. S2: Characterization of the cell type classification task. Fig. S3: Random projection models as baseline models for cell embedding models. Fig. S4 Saliency-based interpretation of models trained on human kidney. [file 13059_2021_2452_MOESM1_ESM.pdf]

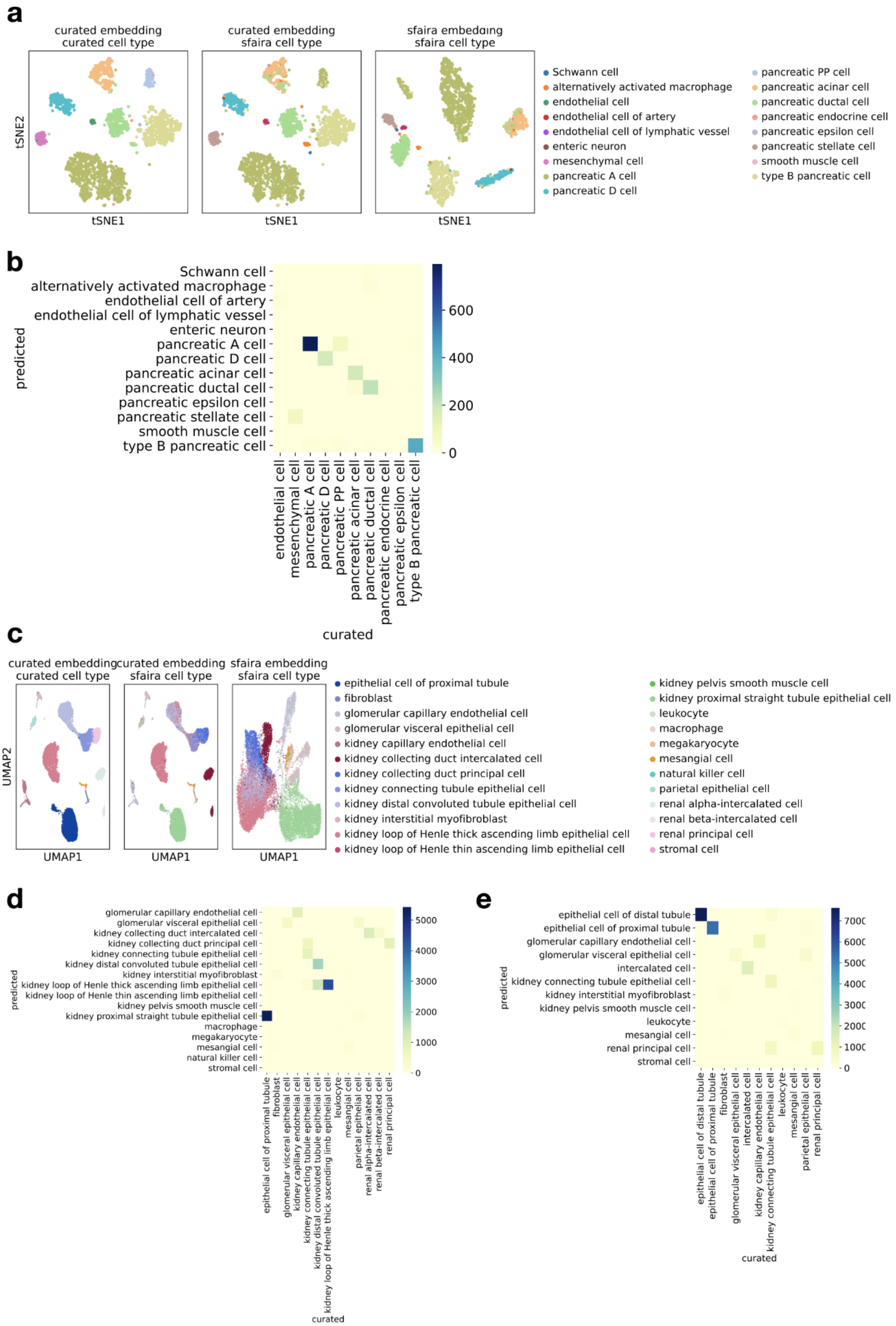
a
curated embedding
curated cell type
curated embedding
sfaira cell type
sfaira embedding
sfaira cell type
tSNE2
tSNE1
Schwann cell
alternatively activated macrophage
endothelial cell
endothelial cell of artery
endothelial cell of lymphatic vessel
enteric neuron
mesenchymal cell
pancreatic A cell
pancreatic D cell
pancreatic PP cell
pancreatic acinar cell
pancreatic ductal cell
pancreatic endocrine cell
pancreatic epsilon cell
pancreatic stellate cell
smooth muscle cell
type B pancreatic cell
b
predicted
Schwann cell
alternatively activated macrophage
endothelial cell of artery
endothelial cell of lymphatic vessel
enteric neuron
pancreatic A cell
pancreatic D cell
pancreatic acinar cell
pancreatic ductal cell
pancreatic epsilon cell
pancreatic stellate cell
smooth muscle cell
type B pancreatic cell
600
400
200
0
endothelial cell
mesenchymal cell
pancreatic A cell
pancreatic D cell
pancreatic PP cell
pancreatic acinar cell
pancreatic ductal cell
pancreatic endocrine cell
pancreatic epsilon cell
type B pancreatic cell
curated
c
curated embedding
curated cell type
curated embedding
sfaira cell type
sfaira embedding
sfaira cell type
UMAP2
UMAP1
epithelial cell of proximal tubule
fibroblast
glomerular capillary endothelial cell
glomerular visceral epithelial cell
kidney capillary endothelial cell
kidney collecting duct intercalated cell
kidney collecting duct principal cell
kidney connecting tubule epithelial cell
kidney distal convoluted tubule epithelial cell
kidney interstitial myofibroblast
kidney loop of Henle thick ascending limb epithelial cell
kidney loop of Henle thin ascending limb epithelial cell
kidney pelvis smooth muscle cell
kidney proximal straight tubule epithelial cell
leukocyte
macrophage
megakaryocyte
mesangial cell
natural killer cell
parietal epithelial cell
renal alpha-intercalated cell
renal beta-intercalated cell
renal principal cell
stromal cell
d
predicted
glomerular capillary endothelial cell
glomerular visceral epithelial cell
kidney collecting duct intercalated cell
kidney collecting duct principal cell
kidney connecting tubule epithelial cell
kidney distal convoluted tubule epithelial cell
kidney interstitial myofibroblast
kidney loop of Henle thick ascending limb epithelial cell
kidney loop of Henle thin ascending limb epithelial cell
kidney pelvis smooth muscle cell
kidney proximal straight tubule epithelial cell
macrophage
megakaryocyte
mesangial cell
natural killer cell
stromal cell
5000
4000
3000
2000
1000
0
epithelial cell of proximal tubule
fibroblast
glomerular visceral epithelial cell
kidney capillary endothelial cell
kidney connecting tubule epithelial cell
kidney distal convoluted tubule epithelial cell
kidney loop of Henle thick ascending limb epithelial cell
leukocyte
mesangial cell
parietal epithelial cell
renal alpha-intercalated cell
renal beta-intercalated cell
renal principal cell
curated
e
predicted
epithelial cell of distal tubule
epithelial cell of proximal tubule
glomerular capillary endothelial cell
glomerular visceral epithelial cell
intercalated cell
kidney connecting tubule epithelial cell
kidney interstitial myofibroblast
kidney pelvis smooth muscle cell
leukocyte
mesangial cell
renal principal cell
stromal cell
7000
6000
5000
4000
3000
2000
1000
0
epithelial cell of distal tubule
epithelial cell of proximal tubule
fibroblast
glomerular visceral epithelial cell
intercalated cell
kidney capillary endothelial cell
kidney connecting tubule epithelial cell
leukocyte
mesangial cell
parietal epithelial cell
renal principal cell
curated

(Figure continues)

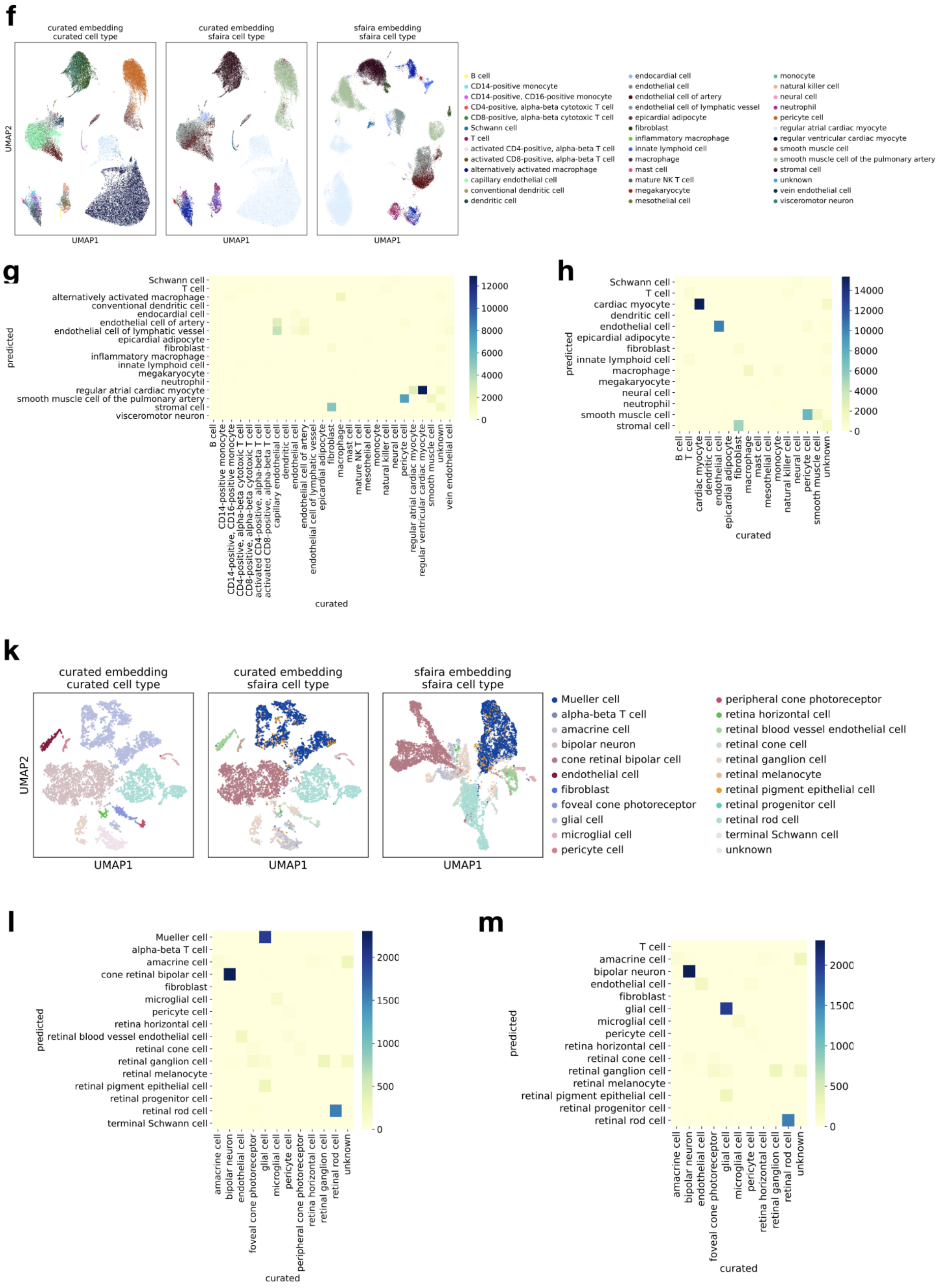

Fig. S1: Zero-shot analysis example cases. Shown are summaries of four zero-shot analysis scenarios which were not included in the training and testing data of the sfaira model zoo presented here (Methods): A pancreas data set **(a, b)**, a kidney data set **(c-e)**, a heart data set **(f-h)**, and a eye data set **(k-m)**. Shown are UMAPs of either the curated embedding published by the authors or a UMAP of a sfaira embedding model with curated cell types superimposed and with sfaira predicted cell types superimposed **(a,c,f,k)**. Note that predicted and curated cell types are not always the same but often stand in an

ontological relationship, which derives from annotation coarsity differences between model training data and these new data sets. The relation between these predicted and observed cell types is further characterised in confusion matrices **(b,d,g,l)**, which were simplified to coarser cell ontology parent nodes in **(e,h,m)**. The analyses presented here were performed after the final fitting of the models that are also available on zenodo and thus represent the true behaviour of this system on new data. Minor inaccuracies in prediction are likely due to insufficient training data annotation or are related to issues in generalisation to the unseen domain effects in these test cases and can be resolved quickly in secondary analyses following this preliminary analysis.

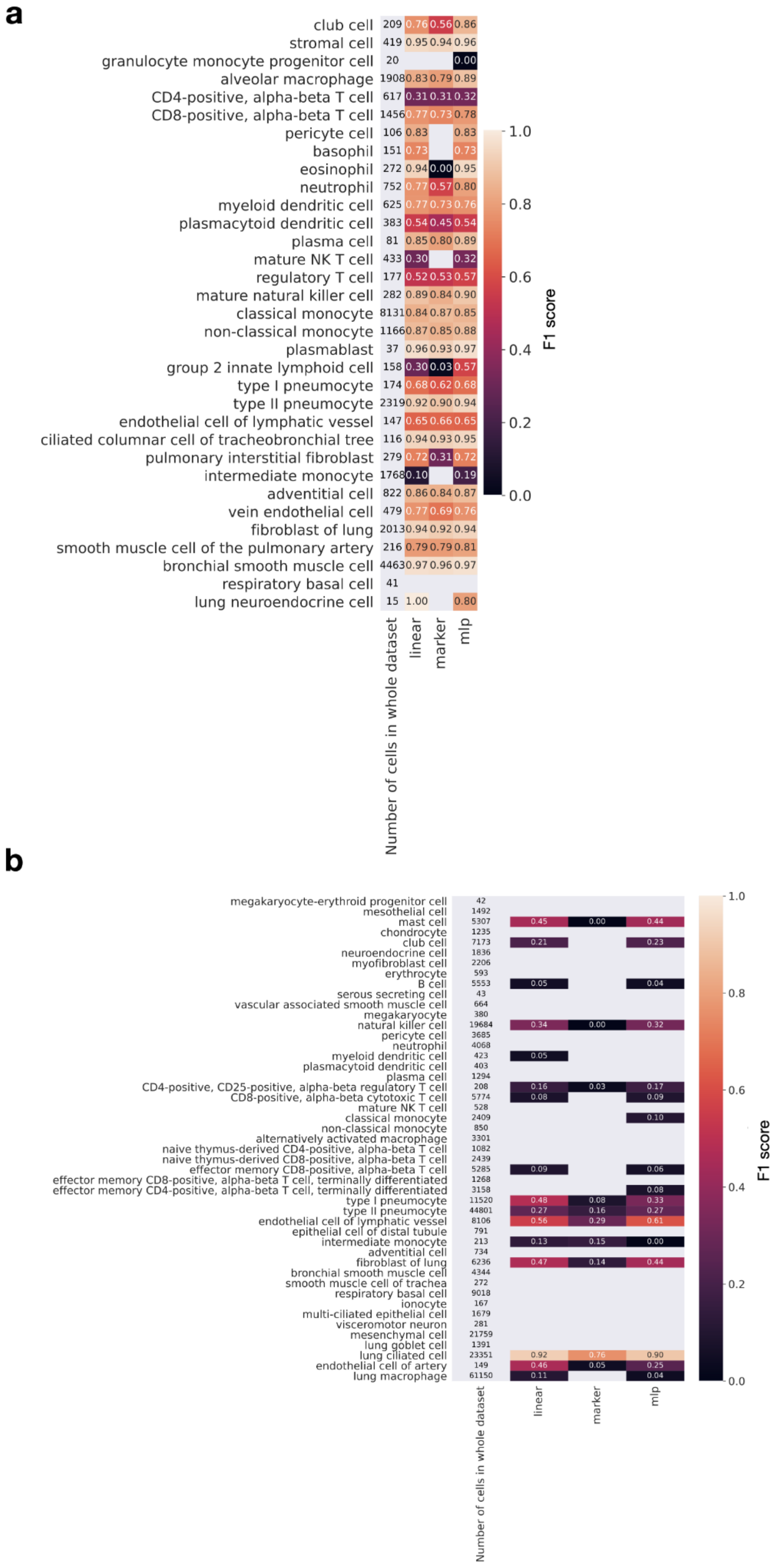

Fig. S2: Characterization of the cell type classification task. (a,b) Cell type wise accuracies for lung samples from humans **(a)** and mice **(b).**

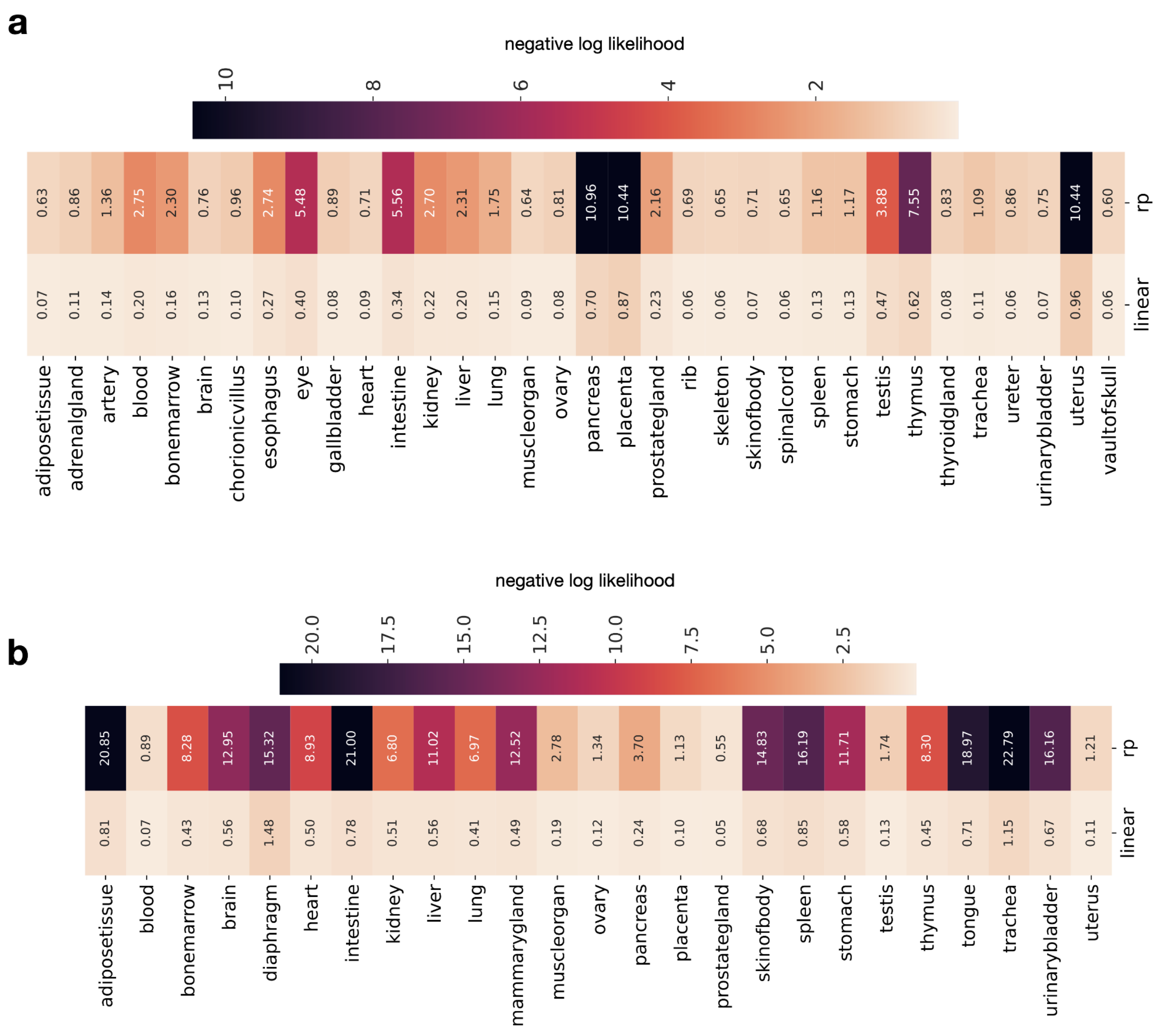

Fig. S3: Random projection models as baseline models for cell embedding models. Shown are the negative log likelihood on the held out test data of a linear embedding model as Fig. 5, and a random projection model (Methods) for human **(a)** and mouse **(b)**. Across all organs, the random projection model had significantly lower negative log likelihoods than the linear model when comparing the mean performance across cross validations in a one-sided paired t-test (human: p=1.11e-05, mouse: p=2.11e-07).

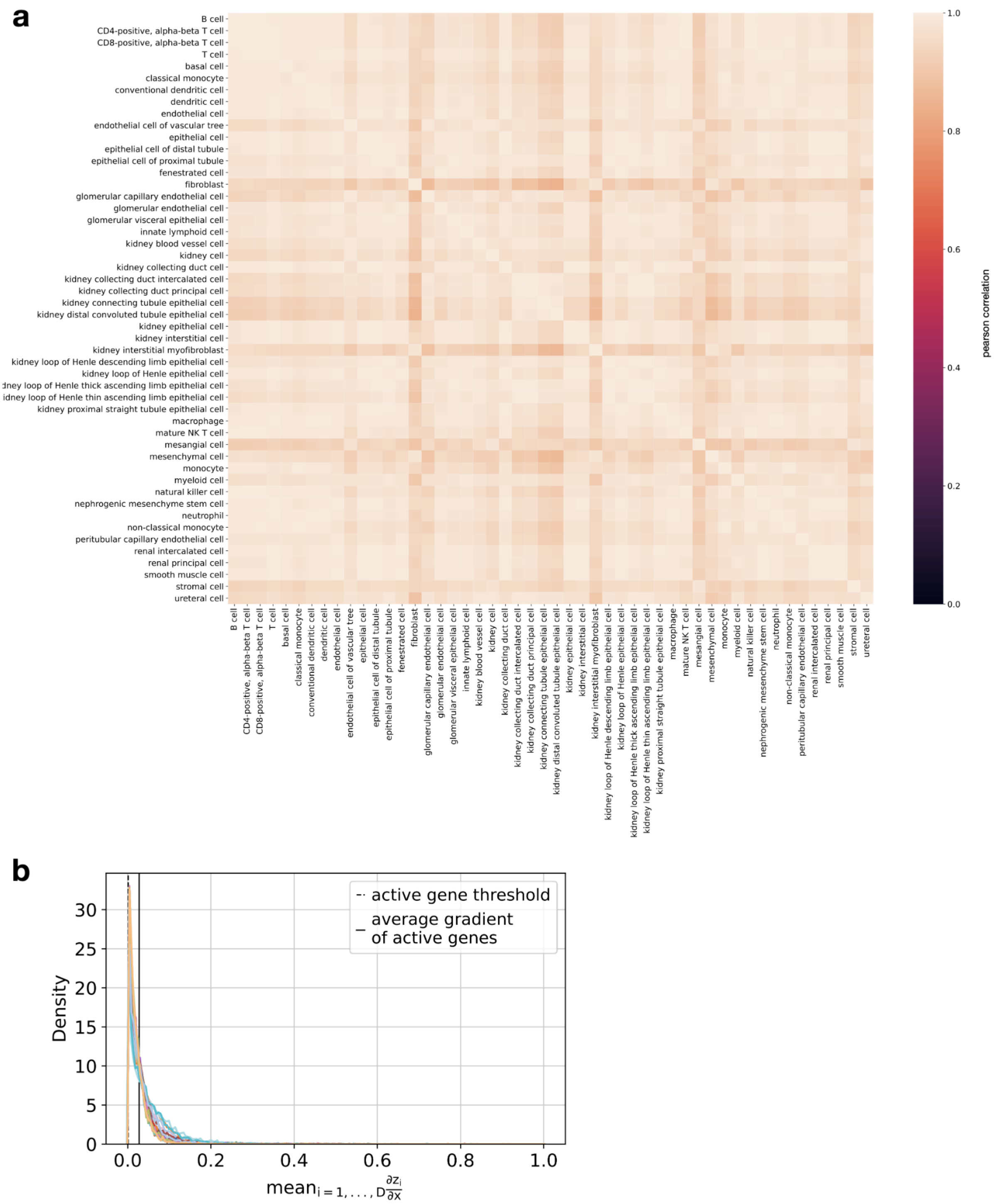

Fig. S4 Saliency-based interpretation of models trained on human kidney. Shown are results for an autoencoder. **(a)** Correlation of cell-type wise aggregated gradients of embedding with respect to input features. **(b)** Distribution of feature-wise wise aggregated gradients of embedding with respect to input features by cell type (color).
