# Supplementary material for: Sfaira accelerates data and model reuse in single cell genomics
Source: Genome Biol. 2021 Aug 25;22:248. doi: 10.1186/s13059-021-02452-6 (PMC8386039; doi:10.1186/s13059-021-02452-6)
Supplement: Supplementary file 2 — Additional file 2. Review history. [file 13059_2021_2452_MOESM2_ESM.docx]

Review History

**First round of review**

**Reviewer 1**

**Were you able to assess all statistics in the manuscript, including the appropriateness of statistical tests used?**

There are no statistics in the manuscript.

**Were you able to directly test the methods?**

No

**Comments to author:**

Summary

Fischer, Dony et al. describes sfaira, a data and model zoo for single cell RNA-seq genomic data that allows standardized sharing of analysis pipelines/models. With the exception of log-normalization of counts, sfaira presents ready to use models on 233 single cell RNA-seq datasets. The authors present different embedding and cell-type annotation models but note that sfaira is not limited to these models. I believe sfaira is a much-needed development in for the single cell community that can truly help with standardizing analysis steps and allow easy application of computational methods to large datasets, directly facilitating the development of reproducible and generalizable methods. While I am generally positive, in regards to the idea and the implementation, I have some remarks which I would like the authors to address.

Major comments

1. There are key details missing from the methods section. For example, I could not find a description of the gene-centric cell-type annotation model in the Methods. Also, the loss function is only described textually, I would appreciate adding the formulas. Similarly, briefly describe the architectures of the AEs and VAEs (now only in the code).

2. In relation to the previous point, I was not able to check the code used to generate the results since the Github (https://github.com/theislab/sfaira_benchmarks) is inaccessible. Also, the accompanying website lacks important details regarding installation and usage so I could not try it out myself.

3. To deal with the different annotations of different datasets, the authors propose a unified ontology. As I understand, this ontology is built manually with guidance from The Cell Ontology. This is perhaps the biggest limitation of sfaira in my opinion since the construction of the ontology is very challenging. I could only find few ontologies on the sfaira website but I assume those were built for all tissues. Perhaps the authors can elaborate on this and discuss the limitations.

4. Do I understand correctly that models are defined per tissue and per species? Cross-species analysis is of great interest to many researchers. Can sfaira be used in this case, for instance to assess how well human cells map to mouse cells?

5. I appreciate the authors reporting of the reconstruction error of the AE to quantitatively assess the quality of the embedding. I find it however difficult to interpret these numbers. As in, assuming a terrible embedding, how big would this loss be compared to the 0.14 obtained for the PBMC data (Fig.3b)? Can you put this number in context?

6. The automated prediction is very coarse (Fig.3b). I am not sure I understand why that is the case. The authors state that with further additions to the data zoo, this will improve. But existing datasets certainly have deeper annotations that can be used for training. Or is sfaira limited to giving annotations at the highest level possible (given the training data)?

7. Based on Fig.4b and c, the authors concluded that all three cell-type prediction models performed well. From the figures, it seems that the marker gene-based classifier has always lower performance and in some cases quite bad performance (e.g. mouse pancreas). Based on previous observations by Abdelaal, Michielsen et al (https://doi.org/10.1186/s13059-019-1795-z) and the presented results it seems that marker-based classifiers are not to be preferred.

8. The performance of cell-type prediction models is significantly different between mouse and human. Why is that. Is it dependent on the amount of training data available?

9. I very much like the idea put forward to use large reference databases as a way to regularize models. Is there any evidence from the models trained in Sfaira that this is the case? As in, if you train on one dataset, the model overfits, but if you train on 10 then there is no overfitting anymore.

10. Throughout the manuscript, the authors propose sfaira as a zoo for single cell genomic data in general. However, only RNA-seq examples are presented. While I believe the concept can be extended, there are few considerations to be taken into account before it is applicable to other data types (for example, different normalization).

Minor comments

1. Sentence not clear "Such a prediction can be achieved with a softmax transform of the output states i n the layer, the loss can be evaluated based on cross-entropy loss."

2. Installation instruction are very brief. Tutorials inaccessible.

3. In Fig.3a, I believe "normalization" should also be added as a step in the sfaira workflow.

4. The legend for Fig.4a does not explain what is in the figure. Which are the true labels and which are the predictions?

5. Fig4d and e shows the performance of the 3 models for lung cell-types. I would then assume that for every value on the x-axis (where there is data), that there are three colored points aligned vertically (corresponding to the performance of the 3 models for that particular cell type). That does not seem to be the case.

6. The y-axis labels in Fig4d and e are written differently.

7. The results shown in Fig5e and f are poorly described in the text and the caption.

8. Perhaps my own shortcoming here, but I don't get how Fig.6a or Supp. Fig. 2a show that linear models and autoencoders are similar in the size of feature sets considered important?

**Reviewer 2**

**Were you able to assess all statistics in the manuscript, including the appropriateness of statistical tests used?**

Yes, and I have assessed the statistics in my report.

**Were you able to directly test the methods?**

No

**Comments to author:**

The authors provide a single-cell RNA-seq data analyzing tool, named sfaira, for retrieving public data sets (data zoo) and using pre-trained models (model zoo) for exploratory analysis of scRNA-seq data sets. The idea of retrieving public data and using pre-trained models is interesting. However, there are many issues with the manuscript.

1. As a manuscript about software, reproducibility of the workflow is critical. However, the description of the examples and analyses in this manuscript are often vague and hand waving, lacking of proper practice of reproducibility and rigor. There is not sufficient information for readers to follow the workflow of the proposed software and understand to use it. A good practice is the paper of Kipoi repository, which shows the command line to run their software. The authors is strongly advised to follow the style of Kipoi repository (https://www.nature.com/articles/s41587-019-0140-0).

2. The manuscript mostly describes what were implemented in the sfaira package, but gives little biological insights. Though it shows some analyses results generated by some existing methods implemented in the sfaira, there is no comparison across these methods. It is unclear what the benefit of using sfaira implementation is. The authors is suggested to follow the comparison and presentation in the paper of Kipoi repository.

3. In the analyses, very little interpretation was given to their results. It is often unclear what the point the authors tried to make with their figures. Take Figure 2 as an example. What is the point of Fig 2a? Also, what Fig 2c shows, i.e. positive relationship between the number of cells and the number of identified cell types, is well known (Ref. 1). What is the point to show this? All subfigures in Fig 2 only show the statistics in the current collection. What is the point of 114-120? Isn't this something expected? How does this show the advantage of sfaira?

4. The author proposed a new way to do embedding and cell type annotation. Though Figure 3b shows one demonstration, more examples should be illustrated to show the generality of its superiority.

5. Figure 4: the authors presented a new algorithm for cell type prediction. However, it was not compared with existing methods. It is unclear what the advantage of using sfaira is. In line 429, what is the meaning of positive classes? The notation is confusing without sufficient explanation. What are yk and ynk,? and What is pnk, etc? The explanations for equation (4) in Method is not adequate.

6. The new method described in Figure 5 is actually similar to the cell type prediction in (Ref. 2). For hold-one-data-set-out test, what is the Negative log likelihood? How was it calculated? How many data sets were used for each organ? Based on Fig 1b, it seems that there were less than 5 data sets. Given the small sample size, the reliability for this test may be questionable. For example, in the Figure 5a/b, the authors claim a clear cell type separation, but why the likelihood in Figure 5c/d for pancreases is quite different (>1 for human, 0.35 for mouse)? The explanation of Figure 5 is insufficient.

7. Also, many citations are not peer-viewed publications, such as citation 1, 10, 11, 17, 18. So the supporting evidence in current manuscript is weak.

8. Finally, about the computation, the authors use the language of "a few commands", "a few lines" many times in the manuscript, without any specifics. Also a computational time comparison is needed to show that Sfaira outperforms other existing tools.

9. In line 146-147, it says "this analysis is entirely automated through a few lines of code, thereby reducing the time required to explore the data by a few hours or even days, depending on the expertise and domain knowledge of the analyst." This is very hand waving. How much time is exactly saved? If the method is reproducible, how can it be dependent on the analyst's expertise and knowledge level? An objective quantification is needed.

Typos,

Line 22, und -> and

Line 164 theislab?

Line 201 Supp Fig 12?

Ref.1 Svensson, Valentine, Eduardo da Veiga Beltrame, and Lior Pachter. "A curated database reveals trends in single-cell transcriptomics." Database 2020 (2020).

Ref. 2 Stuart, Tim, et al. "Comprehensive integration of single-cell data." Cell 177.7 (2019): 1888-1902.

**Authors Response**

**Point-by-point responses to the reviewers’ comments:**

Reviewer #1 (Remarks to the Author): Summary

Fischer, Dony et al. describes sfaira, a data and model zoo for single cell RNA-seq genomic data that allows standardized sharing of analysis pipelines/models. With the exception of log-normalization of counts, sfaira presents ready to use models on 233 single cell RNA-seq datasets. The authors present different embedding and cell-type annotation models but note that sfaira is not limited to these models. I believe sfaira is a much-needed development in for the single cell community that can truly help with standardizing analysis steps and allow easy application of computational methods to large datasets, directly facilitating the development of reproducible and generalizable methods. While I am generally positive, in regards to the idea and the implementation, I have some remarks which I would like the authors to address.

*We thank the reviewer for this positive evaluation and especially for the feedback on cell ontologies. We addressed all remarks as outlined below.*

Major comments

1. There are key details missing from the methods section. For example, I could not find a description of the gene-centric cell-type annotation model in the Methods. Also, the loss function is only described textually, I would appreciate adding the formulas. Similarly, briefly describe the architectures of the AEs and VAEs (now only in the code).

*Indeed, these architectures were before only described in the code; we now extended the method section with the model topologies.*

*L. 466: An example multilayer perceptron for cell type prediction used in this study was trained on all protein coding genes from either mouse or human, had one hidden layer of the size (128), was trained without L1 and L2 penalties on the parameters, and with a selu activation function.*

...

*We defined a marker gene-dominated model to predict cell types from gene expression data. In this model, a sigmoid function based on a gene-specific linear embedding of the gene expression values models an expression threshold. After this gene-wise embedding, a fully connected layer pools information from all genes to the cell type prediction.*

*...*

*An example autoencoder used in this study was trained on all protein coding genes from either mouse or human, had three hidden layers of the sizes (512, 64, 512), was trained without L1 and L2 penalties on the parameters and without input drop-out, was trained with batchnorm between dense layers, with selu activation function, and with a single trained dispersion parameter per gene in the output for the negative binomial reconstruction loss.*

...

*An example variational autoencoder used in this study was trained on all protein coding genes from either mouse or human, had three hidden layers of the sizes (512, 64, 512), was trained without L1 and L2 penalties on the parameters and without input drop-out, was trained with batchnorm between dense layers, with selu activation function, and with a single trained dispersion parameter per gene in the output for the negative binomial reconstruction loss.ise embedding, a fully connected layer pools information from all genes to the cell type prediction.*

*We also added a description of the random projection method used as a baseline model in the new version of this manuscript. We now added the loss functions for embedding models into the methods section:*

*L. 428: The negative log likelihood over N samples and J genes is:*

*where µ is the mean and φ the dispersion parameter of a negative binomial distribution. We would like to point out that it is very easy to switch loss functions in sfaira but we agree that writing these cost functions down is useful to the reader to understand the benchmarks performed in this manuscript.*

2. In relation to the previous point, I was not able to check the code used to generate the results since the Github (https://github.com/theislab/sfaira_benchmarks) is inaccessible. Also, the accompanying website lacks important details regarding installation and usage so I could not try it out myself.

*Thanks for your interest and sorry for the confusion - the reproducibility code was previously attached as a supplementary data item. We now published it on GitHub under: https://github.com/theislab/sfaira_benchmarks. Moreover, we published extensive tutorials https://github.com/theislab/sfaira_tutorials and improved the code documentation https://sfaira.readthedocs.io/en/latest/, including installation instructions.*

3.	To deal with the different annotations of different datasets, the authors propose a unified ontology. As I understand, this ontology is built manually with guidance from The Cell Ontology. This is perhaps the biggest limitation of sfaira in my opinion since the construction of the ontology is very challenging. I could only find few ontologies on the sfaira website but I assume those were built for all tissues. Perhaps the authors can elaborate on this and discuss the limitations.

*As the reviewer described, we previously maintained custom cell type ontologies on the sfaira GitHub repository, which made it initially simpler for us to merge annotation across so many data sets. However, we agree (and learnt a bit the hard way) that custom ontologies come with scaling and maintenance problems. In this revision, we therefore as suggested/implied carried our entire data library over to the Cell Ontology, the main cell type ontology consortium which is also used in many Human Cell Atlas efforts. It has to be noted that transferring free text cell type labels to cell ontology labels is very laborious and, therefore, not performed in most published data sets.*

*To incentivise this in the future, we built a semi-automatic label translation tool into sfaira that takes both string-similarity and anatomic location into account. There is also the issue of Cell Ontology versions when public releases are used: Cell Ontology releases every few weeks and there is no single ontology that is constant in time. To this end, we built an interface from sfaira directly to Cell Ontology releases to be able to query specific versions.*

*Together, this solution to cell type handling significantly improves model fitting based on ontology structured cell type labels in sfaira. This cell ontology brought a major increase in complexity of the directed acyclic graph that connects cell types; we therefore re-trained all cell type classifier models. The results are consistent, and are presented in Fig. 4.*

4.	Do I understand correctly that models are defined per tissue and per species? Cross-species analysis is of great interest to many researchers. Can sfaira be used in this case, for instance to assess how well human cells map to mouse cells?

*It is correct that we trained models per tissue and species, and hence label transfer was not easily possible. During this revision motivated by this comment, we changed the way data can be accessed as we now represent tissues within the UBERON tissue ontology. Accordingly, training data can be queried for very specific anatomic locations or large organs. In this revision, we still trained models on commonly sampled anatomic locations for these to be useful in common scenarios. UBERON is a cross-species ontology which allows us to query tissues such as “lung” across species without any problems. It has to be noted that the feature space is different between organisms: genes have to be matched via orthologues. We have not introduced orthologue mapping yet (or more complex ways of cross-species translation), but this can be done easily based on the data matrices that we produce with external gene name conversion tools. Importantly, the new cell type ontology is also cross-species so that also cell type predictors could be fit across species. In summary, sfaira provides a great current infrastructure to prepare cross-species model fits but such (new) models are not benchmarked here.*

5.	I appreciate the authors reporting of the reconstruction error of the AE to quantitatively assess the quality of the embedding. I find it however difficult to interpret these numbers. As in, assuming a terrible embedding, how big would this loss be compared to the 0.14 obtained for the PBMC data (Fig.3b)? Can you put this number in context?

*Good point. We added a random projection (https://scikit-learn.org/stable/modules/random_projection.html) as a baseline embedding model into this analysis to contextualise the metric magnitudes, comparing with the linear model fits (Supp. Fig. 3). The random projection is much worse in almost all cases, showing that while the presented models work similarly well, they work much better than bad models. To quantify deviation from the random model, we computed one-sided p-values from a paired t-test on the mean negative log likelihood of both linear and random projection model, both in human and mice these tests were significant at a p-value threshold of 1e-3 (human: p=1.11e-05, mouse: p=2.11e-07).*

*L. 347: Supp. Fig. 3: Random projection models as baseline models for cell embedding models. Shown are the negative log likelihood on the held out test data of a linear embedding model as Fig. 5, and a random projection model (Methods) for human (a) and mouse (b). Across all organs, the random projection model had significantly lower negative log likelihoods than the linear model when comparing the mean performance across cross validations in a one-sided paired t-test (human: p=1.11e-05, mouse: p=2.11e-07).*

6. The automated prediction is very coarse (Fig.3b). I am not sure I understand why that is the case. The authors state that with further additions to the data zoo, this will improve. But existing datasets certainly have deeper annotations that can be used for training. Or is sfaira limited to giving annotations at the highest level possible (given the training data)?

*The predictions shown in Fig. 3b are for human PBMC. The coarsity only depends on the coarsity of the training data, which was relatively coarse in the last version of our data library: For the model corresponding to each anatomic partition, we defined the label space as the set of most fine grained labels which were observed in the training data. Therefore, the models become naturally more fine grained as the data universe expands. We included more PBMC data sets in this revision and could achieve more fine grained labels in this round (Fig. 3b). Importantly, the training mechanisms associated with using cell ontology-constrained data naturally scales the coarseness of cell type models with increasing availability of data across the entire data set collection.*

*L. 296: Figure 3: Sfaira automatizes exploratory analysis of single-cell data. (a) Manual single-cell data analysis pipeline and automated sfaira pipeline. (b) Comparison of manual feature engineering workflow with automated embedding and cell type annotation from sfaira on human PBMC dataset. Shown are UMAPs based on a PCA of an engineered feature space and of the out-of-the-box latent space from a linear sfaira embedding model. Superimposed are cell types previously annotated for this data set and sfaira cell type predictions.*

7.	Based on Fig.4b and c, the authors concluded that all three cell-type prediction models performed well. From the figures, it seems that the marker gene-based classifier has always lower performance and in some cases quite bad performance (e.g. mouse pancreas). Based on previous observations by Abdelaal, Michielsen et al (https://doi.org/10.1186/s13059-019-1795-z) and the presented results it seems that marker-based classifiers are not to be preferred.

*The marker model proposed here was similar in performance to the linear and non-linear models in most organs and only performed worse in few cases. Prior knowledge was not yet included in these models, they are merely architectures that allow for easy integration of prior knowledge about marker genes. We included this finding explicitly into the text, citing the above source on marker-based models:*

*L. 182: All models performed well as expected based on previous findings24 on selected organs (Fig. 4b,c), with a median accuracy of 0.64 in human samples and 0.93 in mouse samples. We did not find performance advantages of the marker model.*

8.	The performance of cell-type prediction models is significantly different between mouse and human. Why is that is it dependent on the amount of training data available?

*Many mouse samples come from tabula muris senis (Tabula Muris Consortium 2020) and the mouse cell atlas (Han et al. 2018). The tabula muris senis datasets were split into multiple samples for each tissue, based on mouse age and single-cell technology (10x Sequencing and SMART-Seq2). The mouse cell atlas data already exist in high numbers of samples (biological replicates) for many tissues. Accordingly, the test sets come from data that is similar to the training data in terms of experimental procedure and annotation. This is most likely a main reason for the performance of the mouse classifiers to be stronger than in the human case, where data sets often come from different studies. Additionally, anatomic partitions in which we only had access to a single study, ie. where study hold-out was not possible, were split into train and test data by randomly sampling cells, which also results in relatively easy test splits, we add this methodology in the methods:*

*L. 514: Test data splits: Where available, entire data sets were held-out to evaluate model performance test metrics. Some organs were only represented by a single data set in the data zoo. In these cases, we held out a random set of 20% of all cells as test data.*

*We note that this is not a main finding of the paper and that classifiers will become more stable as the data universe expands. We added this discussion of model performance as a function of data availability into the discussion:*

*L. 274: The models proposed for automated data analysis are likely to improve drastically with increasing availability of training data resulting in strong performance improvements in the near future as the data zoo grows.*

9.	I very much like the idea put forward to use large reference databases as a way to regularize models. Is there any evidence from the models trained in Sfaira that this is the case? As in, if you train on one dataset, the model overfits, but if you train on 10 then there is no overfitting anymore.

*Data regularization is typically observed in other areas of Deep Learning that such regularization takes place and it is a general applied statistics concept underlying the mathematics of overfitting. Benchmarking abstraction and interpretability of embeddings is, however, a large effort and after some discussion we decided out of scope for this manuscript. We improved the discussion of this topic in the results:*

*L. 227: In principle, models can also be regularized through extremely diverse training data, thus making it hard for the model to overfit the entire training domain and forcing the model to learn strong abstractions. Importantly, this data-driven approach stands in contrast to the usage of variance explaining covariates in conditional embedding models: An embedding model with a high degree of abstraction should be able to learn abstract representations of gene expression configurations across conditions, similar to how image-trained convolutional networks learn representations of images from different objects or sources without having access to categorical descriptors of these conditions. Conditional embedding models are often used in data integration studies, in which domain differences are usually removed by a projection mechanism28. Sfaira provides structured data libraries built for providing models with extremely large training data sets. Indeed, we could converge embedding models on such large data zoos of scRNA-seq data of whole organisms across datasets from many studies (Fig. 5e,f). In summary, sfaira is well positioned to enable model regularization through, extremely diverse training data sets, with the aim of extending reference data usage from projection-based data integration to more abstract pre-trained embeddings.*

10.	Throughout the manuscript, the authors propose sfaira as a zoo for single cell genomic data in general. However, only RNA-seq examples are presented. While I believe the concept can be extended, there are few considerations to be taken into account before it is applicable to other data types (for example, different normalization).

*We entirely agree that more data modalities will fit into our framework. We are already working on adaptation to CITE-seq, spot-based transcriptomics, scATAC-seq and other commonly used modalities. We added this to the discussion:*

*L. 269: Moving beyond scRNA-seq, we will support different data modalities such as from splicing annotation, scATAC-seq34, CITE-seq31 and spatial molecular profiling in the near future.*

*We aim to supply as raw data as possible, so in many cases normalisation will be a downstream choice that is not influenced by sfaira data loaders.*

Minor comments

1.	Sentence not clear "Such a prediction can be achieved with a softmax transform of the output states i n the layer, the loss can be evaluated based on cross-entropy loss."

*We re-phrased this sentence to:*

*L. 434: The loss typically used for evaluating fits of such probability mass distributions is the cross-entropy loss.*

2.	Installation instruction are very brief. Tutorials inaccessible.

*We improved the installation instructions here LINK. Tutorials are accessible here https://github.com/theislab/sfaira_tutorials.*

3.	In Fig.3a, I believe "normalization" should also be added as a step in the sfaira workflow.

*The reviewer is right in pointing out that there are normalisation layers in our models. However, this is a modelling choice and may be solved differently in other modelling projects. Moreover, this normalisation is not subject to typical considerations in normalization (Luecken and Theis 2019), it simply scales cell-wise vectors in the model input and therefore does not present a “branching point” in analysis pipelines, as it does in a standard workflow in which normalisation methods have to be carefully chosen to match the full pipeline.*

4.	The legend for Fig.4a does not explain what is in the figure. Which are the true labels and which are the predictions?

*We added the description in the caption and believe that this panel is more accessible now:*

*L. 304: The y axis contains leaf nodes of a cell type ontology, which can be combined linearly to yield the predicted probability mass of any other node in the ontology graph (x axis).*

5.	Fig4d and e shows the performance of the 3 models for lung cell-types. I would then assume that for every value on the x-axis (where there is data), that there are three colored points aligned vertically (corresponding to the performance of the 3 models for that particular cell type). That does not seem to be the case.

*Indeed, one would expect sets of three points per x value. The missing points coincide with performance measures from other models and thus lie on top of each other. We reduced opacity in the new version of this plot. Note that we supplied the linear extrapolation to mitigate such visualisation issues.*

6.	The y-axis labels in Fig4d and e are written differently.

*We addressed this mistake and would like to thank the reviewer for pointing us to this mistake.*

7.	The results shown in Fig5e and f are poorly described in the text and the caption.

*We improved the caption and the discussion of Fig. 5 in the results:*

*l. 217: Data integration is a trade-off between removing between-sample variance resulting from technical effects and conserving biologically meaningful variance3. Instead of removing between-sample variance in a data integration setting, we focus on embedding models that discover axes of variation which allow us to discern biological variation on a new data set (zero-shot learning17). Here, it is difficult to discern models that overfit all variation in data sets and models that capture only relevant axes of variation. This overfitting is an issue that can be addressed through regularization. Model regularization in embedding and cell type classifier neural networks is often performed via L1 or L2 constraints on model parameters, via drop-out mechanisms and via dimension bottlenecks in latent representations. While effective in the prevention of overfitting, these regularization methods cannot be easily used to derive interpretable models. Instead, they dynamically limit the degrees of freedom of generously over-parameterized models.*

*In principle, models can also be regularized through extremely diverse training data, thus making it hard for the model to overfit the entire training domain and forcing the model to learn strong abstractions. Importantly, this data-driven approach stands in contrast to the usage of variance explaining covariates in conditional embedding models: An embedding model with a high degree of abstraction should be able to learn abstract representations of gene expression configurations across conditions, similar to how image-trained convolutional networks learn representations of images from different objects or sources without having access to categorical descriptors of these conditions. Conditional embedding models are often used in data integration studies, in which domain differences are usually removed by a projection mechanism. Sfaira provides structured data libraries built for providing models with extremely large training data sets. Indeed, we could converge embedding models on such large data zoos of scRNA-seq data of whole organisms across datasets from many studies (Fig. 5e,f). In summary, sfaira is well positioned to enable model regularization through and training on extremely diverse training data sets, with the aim of extending reference data usage from projection-based data integration to more abstract pre-trained embeddings.*

8. Perhaps my own shortcoming here, but I don't get how Fig.6a or Supp. Fig. 2a show that linear models and autoencoders are similar in the size of feature sets considered important?

*Indeed, this finding was not well characterised, we re-phrased this to:*

*L. 246: Moreover, we found that linear models and autoencoders are similar in the size of feature sets considered important by these gradient-based mechanisms for each cell type (Fig. 6a, Supp. Fig. 2a) and also have a similarly shaped marginal distribution of normalised gradients (Fig. 6b, Supp. Fig. 2b).*

*Note that our main message here was that both show a peak at very low values, in contrast to a multi-modal distribution for example. As with many other analyses in the manuscript, this analysis also shows the feasibility of such an approach in our framework.*

Reviewer #2 (Remarks to the Author):

The authors provide a single-cell RNA-seq data analyzing tool, named sfaira, for retrieving public data sets (data zoo) and using pre-trained models (model zoo) for exploratory analysis of scRNA-seq data sets. The idea of retrieving public data and using pre-trained models is interesting. However, there are many issues with the manuscript.

*We thank the reviewer for general interest and the critical and useful recommendations, which we addressed as discussed below.*

1.	As a manuscript about software, reproducibility of the workflow is critical. However, the description of the examples and analyses in this manuscript are often vague and hand waving, lacking of proper practice of reproducibility and rigor. There is not sufficient information for readers to follow the workflow of the proposed software and understand to use it. A good practice is the paper of Kipoi repository, which shows the command line to run their software. The authors is strongly advised to follow the style of Kipoi repository (https://www.nature.com/articles/s41587-019-0140-0).

*We identify two forms of reproducibility touched upon here: Reproducibility of our benchmarks and reproducible model execution across platforms.*

*To make our benchmarks more transparent and reproducible, we extended the documentation of the reproducibility repository (https://github.com/theislab/sfaira_benchmarks). This repository contains the full set of python scripts and shell scripts used to run grid searches presented in this paper. We also improved tutorials*

*(https://github.com/theislab/sfaira_tutorials)	and	documentation	of	the	API*

*(https://sfaira.readthedocs.io/en/latest/).*

*To address the model execution part we want to first thank the reviewer for raising the link to kipoi. Of course we were inspired in many places by kipoi when designing sfaira, but we think it is important to point out that in many ways, kipoi deals with different problems in functional genomics from the problems we encounter in single-cell biology. Importantly, single-cell analysis workflows are usually more interactive, run on a shorter time scale per step and are commonly performed as markdown documents in R or jupyter notebooks in python. For software like sfaira to be useful, it crucially has to fit into such workflows and we do not believe that a model execution command line interface (as for example in kipoi) would be widely adopted. Containerisation of model execution as in kipoi is also mostly useful if the model frameworks are very different. Users may simply tie in sfaira in their workflow as latent space estimator, but for the more method oriented ones, we also provide direct links to the models: For these we anticipate many models in single-cell latent representation to be built in TensorFlow or PyTorch as this is the effective standard in the ML community. TensorFlow and PyTorch have their own serialisation functionalities that can replace containers to some degree, which significantly reduces contribution and maintenance burden. We added this argumentation in the discussion section:*

*L. 262: We used model deployment infrastructure from TensorFlow here, similar infrastructure is available from PyTorch, and both are very simple to maintain. One could also think about deploying executable models in Docker images, as is currently done in kipoi for functional genomics data.*

2.	The manuscript mostly describes what were implemented in the sfaira package, but gives little biological insights. Though it shows some analyses results generated by some existing methods implemented in the sfaira, there is no comparison across these methods. It is unclear what the benefit of using sfaira implementation is. The authors is suggested to follow the comparison and presentation in the paper of Kipoi repository.

*We would like to point out that we submitted sfaira as a software paper (which was not the case for kipoi). We explicitly chose this format to focus this manuscript on the presentation of the software infrastructure of this tool, making such a large set of modelling options available. Regarding use cases for large-scale atlas compilation, many recent methods with use cases have been published fully or on biorxiv, with Azimuth and scArches being two of the most prominent ones (TODO reference). So we believe it is very clear to users that accessing many, consistently labeled single cell expression data sets and then having shared models on top of this has strong benefit for standard workflows (as demonstrated in the text). Finally, sfaira does not aim to be a final benchmark in this field, but critically enables fast and efficient benchmarking, as was already used recently(Dony et al. 2020), and we now included this more clearly also in the introduction.*

3.	In the analyses, very little interpretation was given to their results. It is often unclear what the point the authors tried to make with their figures. Take Figure 2 as an example. What is the point of Fig 2a? Also, what Fig 2c shows, i.e. positive relationship between the number of cells and the number of identified cell types, is well known (Ref. 1). What is the point to show this? All subfigures in Fig 2 only show the statistics in the current collection. What is the point of 114-120? Isn't this something expected? How does this show the advantage of sfaira?

*The cited section was previously:*

*L.114-120: We can also consider gene-gene dependencies. Often, one is interested in the correlation of expression between genes to establish regulatory relationships. As an example, we investigated the correlation of two cell-cycle-associated genes, Mcm5 and Pcna (Fig. 2b), which provides a range for their correlation and an estimate of how often these genes are correlated across tissues. This analysis shows a range of correlation coefficients including many high values above 0.5, which establishes the expression of these two genes are correlated in many cell types, which is to be expected, because the cell cycle is a common biological confounder of transcriptomic states.*

*The reviewer criticises that the presentation of results is descriptive in some places, such as above. We now extended the motivation. A key contribution of sfaira is infrastructure for efficient and quick aggregation of data based on metadata queries. This infrastructure allows for many types of exploratory analysis that were previously prohibitively difficult to perform in many cases: For example, cell type specific queries (Fig. 2d) across data sets suffered from a lack of consistently annotated cell types and frameworks capable of performing relational reasoning in cell type ontologies. Hence, we used Fig 2 to showcase some such analysis to motivate streams of analyses that can now be routinely performed. Similarly, aggregation of cells across single-cell technology (Fig. 2e,f,g). Similarly, gene-based queries require a harmonised gene space which we facilitate in sfaira, otherwise queries for specific genes, such as insulin (Fig. 2a), are complicated by different gene name conventions and exclusion of genes from processed count matrices in published data sets. The metadata ontologies added in this revision further improve exploratory analysis as shown in Fig. 2 significantly. Data collection-wide gene expression summaries and queries are a huge selling point of cellular atlasses and are made available across “atlas projects” across organisms and tissues in sfaira. We updated the discussion of Fig. 2 in the results:*

*L. 97: Scalable access to data with unified annotations allows for queries of gene and data statistics Streamlined access to unified, large, cross-study data sets as provided by sfaira allows for easy data statistics queries that can be helpful for putting observations in the context of other data sets. The common group of such queries is gene-based. For example, it is often useful to have a reference range for the expression activity of a gene observed with scRNA-seq. This can be done with sfaira via a single straight-forward query, as showcased for Ins1 scaled expression across organs and cell types in mice (Fig. 2a). Note that cell type-wise summary metrics are often much more useful for such workflows than cross-data set averages, which are skewed toward frequent cell types and are more useful than extrema, such as maximum expression, which are heavily influenced by the variance of the expression distribution. This analysis establishes an active range between 0 and 2,500 counts per 10,000 unique molecular identifiers as an active range for Ins1 expression in mice, with all expressing cell types located in pancreas data sets. Next, we consider gene-gene dependencies. Often, one is interested in the correlation of expression between genes to establish regulatory relationships. As an example, we investigated the correlation of two cell-cycle-associated genes, Mcm5 and Pcna (Fig. 2b), which provides a range for their correlation and an estimate of how often these genes are correlated across tissues.*

*A second group of such queries is based on subsetting operations across cells based on cell and dataset metadata. Such queries depend on a homogenous annotation of metadata across data sets. Sfaira enforces this type of annotation by requiring meta data to follow ontologies. In sfaira, we also implemented relational reasoning of metadata items based on ontologies which is often necessary to achieve meaningful subsets. We showcase a few example data zoo summary statistics that exploit metadata-based queries (Fig. 2c-g). We generated complexity plots of the total number of cells versus the number of most fine grained cell type labels per organ to give guidelines for prioritisation of organs for further cell type discovery (Fig. 2c). In a cell type centric scenario, we queried the fraction of T cells across organs (Fig. 2d), a query that can be used to characterise specific cell types across organs and datasets. Last, we queried a summary of total reads per cell summary statistics and protocol summary statistics (Fig. 2e-g).*

4.	The author proposed a new way to do embedding and cell type annotation. Though Figure 3b shows one demonstration, more examples should be illustrated to show the generality of its superiority.

*We did not show conceptually new embedding models but only adapted hyperparameters of models previously described in the literature to our new data libraries: autoencoders (Eraslan et al. 2019), variational autoencoders (Lopez et al. 2018), non-negative matrix factorizations (Stein-O’Brien et al. 2019) and linear models. We included one new cell type prediction algorithm (“marker model”) as an additional reference to show how new models can be easily benchmarked using our tools. Importantly, our results on cell type prediction are largely in agreement with other papers that most reasonable models perform well on this task (Abdelaal et al. 2019; Köhler, Büttner, and Theis 2019): The key finding of our study with respect to cell type predictors is that we can train and deploy cell type models across species and organs to make them useful in a general setting, moving away from example studies that show that models work on a handful of example tissues with manually curated feature spaces tailored for this task. This includes the use of an adapted cross-entropies loss and accuracy metrics which are fundamentally needed to fit classifiers on such data; there is no baseline model for this loss as one cannot fit supervised models on hierarchically structured labels with a standard cross-entropy. We do agree that Fig. 3 is a useful demonstration of how these models and our tools as infrastructure to train such models can be used in the future. To make this demonstration more convincing, we added 4 more such held-out data sets in the new Supp. Fig. 1. This analysis highlights a core advantage of representing data sets through metadata ontologies: Instead of providing a single trained model for blood data only, we present models for 10s of anatomic partitions, covering many commonly assayed organs in mouse and human. As shown in Supp. Fig. 1, this automatic analysis is already good at reproducing previous results resulting from extensive curation of single data sets in dedicated studies. We are able to achieve this performance even though the data collection efforts of sfaira are only in its infancy and our data universe will likely grow considerably in the near future, further improving the power of such models. We compared predicted and observed cell type labels in these test scenarios, mitigating coarsity differences such that the results can still be understood intuitively from confusion matrices (Supp. Fig. 1). Please consider Fig. 4,5 for a more systematic comparison of model performance on held out test data based on aggregate accuracy metrics and negative log likelihood.*

*L. 330: Supp. Fig. 1: Zero-shot analysis example cases. Shown are summaries of four zero-shot analysis scenarios which were not included in the training and testing data of the sfaira model zoo presented here: A pancreas data set (a, b), a kidney data set (c-e), a heart data set (f-h), and a eye data set (k-m). Shown are UMAPs of either the curated embedding published by the authors or a UMAP of a sfaira embedding model with curated cell types superimposed and with sfaira predicted cell types superimposed (a,c,f,k). Note that predicted and curated cell types are not always the same but often stand in an ontological relationship, which derives from annotation coarsity differences between model training data and these new data sets. The relation between these predicted and observed cell types is further characterised in confusion matrices (b,d,g,l), which were simplified to coarser cell ontology parent nodes in (e,h,m). The analyses presented here were performed after the final fitting of the models that are also available from zenodo and thus represent the true behaviour of this system on new data. Minor inaccuracies in prediction are likely due to insufficient training data annotation or are related to issues in generalisation to the unseen domain effects in these test cases and can be resolved quickly in secondary analyses following this preliminary analysis.*

5.	Figure 4: the authors presented a new algorithm for cell type prediction. However, it was not compared with existing methods. It is unclear what the advantage of using sfaira is. In line 429, what is the meaning of positive classes? The notation is confusing without sufficient explanation. What are yk and ynk,? and What is pnk, etc? The explanations for equation (4) in Method is not adequate.

*As established in comment 2.4, we added a “marker model” as a proof-of-concept that new algorithms can be benchmarked with our software. In the last version of this manuscript, we compared this model to other models (linear models and multilayer perceptrons, Fig. 4), which are often used as cell type predictor models in the literature (Köhler, Büttner, and Theis 2019; Abdelaal et al. 2019). We included this comparison again in the new version of Fig. 4 with the new cell ontology labels. The key advances presented here lie in training scalability across anatomic partitions and data sets, and in the usage of cell type labels in ontologies for supervised model training, as discussed in comment 2.4. We improved the description of equation (4,5) (previously 3,4):*

6.	The new method described in Figure 5 is actually similar to the cell type prediction in (Ref. 2). For hold-one-data-set-out test, what is the Negative log likelihood? How was it calculated? How many data sets were used for each organ? Based on Fig 1b, it seems that there were less than 5 data sets. Given the small sample size, the reliability for this test may be questionable. For example, in the Figure 5a/b, the authors claim a clear cell type separation, but why the likelihood in Figure 5c/d for pancreases is quite different (>1 for human, 0.35 for mouse)? The explanation of Figure 5 is insufficient.

*Figure 5 shows embedding models with observed cell types superimposed; this analysis does not constitute a new general method but simply an analysis workflow (Fig. 5a,b) and a benchmark (Fig. 5 c,d) enabled by the sfaira generated models. Such embedding models, for example autoencoders, have been used in many previously published studies (Eraslan et al. 2019). We believe that the reviewer may be hinting at the analysis in Stuart et al. (Ref. 2), in which they used cell type predictors to annotate PBMC data in an online tool “Azimuth”. Sfaira can serve similar purposes but overcomes critical software challenges of Azimuth:*

- *We allow local execution in jupyter notebooks through our python interface instead of requiring interaction with a web interface.*

*-	We use decentralised model zoos, allowing execution of models trained by different entities (ie. not only trained by website maintainers).*

*-	We are not bound to PBMC. The example shown in Fig. 5 could as well be lung, colon or any other organ shown in the rest of the manuscript. A large advantage of sfaira is its flexibility in training and deployment across arbitrary anatomic partitions of data libraries.*

*-	By using cell ontologies, we do not depend on label harmonisation between samples in a training set, making the cell type prediction problems that we can address very general.*

*The negative log likelihood for test data is the same as for training data and is a negative log binomial likelihood, which we chose because it is widely used for scRNA-seq data. We added the negative log binomial likelihood as eq. 1 (embeddings) in the Online methods.*

*The magnitude of the likelihood varies across tissues, this has to do with the amount of technical noise in the data sets used there and with the size of batch effects of the test data with respect to the training data. We added a baseline embedding model (random projection, see also reviewer 1 comment 5) as a reference to help with an intuition for these results. Importantly, the likelihood values are most meaningful within the models fit to one tissue rather than across tissues.*

*We improved the description of Fig. 5 in the main text, see also reviewer 1 comment 7.*

7.	Also, many citations are not peer-viewed publications, such as citation 1, 10, 11, 17, 18. So the supporting evidence in current manuscript is weak.

*We do believe that pre-prints are an integral part of result communication in science and would like to attribute the contributions that these authors made and use these also to characterise trends in the field. We would like to point out that no crucial statements in our study depend on these references alone. We updated preprints where available, and would expect some of those texts to be published.*

8.	Finally, about the computation, the authors use the language of "a few commands", "a few lines" many times in the manuscript, without any specifics. Also a computational time comparison is needed to show that Sfaira outperforms other existing tools.

*We do not claim at any point in the manuscript that we are faster than other tools, for the most part, note also that there are also no tools that can build similar data libraries. In line with the criticism of non-quantified statements on scripting effort, we removed statements such as "a few commands" and "a few lines" throughout the manuscript. We would like to highlight that it is indeed very easy to use sfaira-managed data sets in the context of a data library though, we demonstrate this in the tutorials (https://github.com/theislab/sfaira_tutorials).*

9.	In line 146-147, it says "this analysis is entirely automated through a few lines of code, thereby reducing the time required to explore the data by a few hours or even days, depending on the expertise and domain knowledge of the analyst." This is very hand waving. How much time is exactly saved? If the method is reproducible, how can it be dependent on the analyst's expertise and knowledge level? An objective quantification is needed.

*We apologize for the ambiguity in the sentence cited above, the dependency is in the standard workflow, not in our workflow (which is constant in code and therefore relatively constant in analyst time). It is hard to pinpoint how long a typical analysis workflow takes as it depends on the analysts. Even an optimistic estimate would say a few hours for experts to a few days and more for normal analysts depending on tissue complexity - this compared to minutes with sfaira. So sfaira is the first framework to allow routine execution of exploratory workflows in a general (cross tissue) setting in python, we further demonstrate this in the new tutorials (https://github.com/theislab/sfaira_tutorials) and in the benchmarking code. In line with our response to reviewer 2 comment 8, we removed this sentence.*

Typos,

Line 22, und -> and

*This was a typo and is corrected in the new, shorter abstract. We would like to thank the reviewer for pointing us to this mistake.*

Line 164 theislab?

*This label characterises a model deploying organisation, in this case our GitHub organisation label, and was part of an example model name, syntactically isolated by quotation marks. The mention of this model name was removed when this section was restructured.*

Line 201 Supp Fig 12?

*This reference was meant to point at Supp. Fig. 1, we corrected this mistake. We would like to thank the reviewer for pointing us to this mistake.*

References of reviewer 2:

Ref.1 Svensson, Valentine, Eduardo da Veiga Beltrame, and Lior Pachter. "A curated database reveals trends in single-cell transcriptomics." Database 2020 (2020).

Ref. 2 Stuart, Tim, et al. "Comprehensive integration of single-cell data." Cell 177.7 (2019): 1888-1902.

*References:*

*Abdelaal, Tamim, Lieke Michielsen, Davy Cats, Dylan Hoogduin, Hailiang Mei, Marcel J. T. Reinders, and Ahmed Mahfouz. 2019. “A Comparison of Automatic Cell Identification Methods for Single-Cell RNA Sequencing Data.” Genome Biology 20 (1): 194.*

*Dony, Leander, Martin König, D. Fischer, and Fabian J. Theis. 2020. “Variational Autoencoders with Flexible Priors Enable Robust Distribution Learning on Single-Cell RNA Sequencing Data.” In ICML 2020 Workshop on Computational Biology (WCB) Proceedings Paper. Vol. 37. https://raw.githubusercontent.com/theislab/flexible_priors/master/author_copy.pdf.*

*Eraslan, Gökcen, Lukas M. Simon, Maria Mircea, Nikola S. Mueller, and Fabian J. Theis. 2019. “Single-Cell*

*RNA-Seq Denoising Using a Deep Count Autoencoder.” Nature Communications 10 (1): 390.*

*Han, Xiaoping, Renying Wang, Yincong Zhou, Lijiang Fei, Huiyu Sun, Shujing Lai, Assieh Saadatpour, et al.*

*2018. “Mapping the Mouse Cell Atlas by Microwell-Seq.” Cell 173 (5): 1307.*

*Köhler, N. D., M. Büttner, and F. J. Theis. 2019. “Deep Learning Does Not Outperform Classical Machine*

*Learning for Cell-Type Annotation.” bioRxiv. https://www.biorxiv.org/content/10.1101/653907v1.abstract. Lopez, Romain, Jeffrey Regier, Michael B. Cole, Michael I. Jordan, and Nir Yosef. 2018. “Deep Generative*

*Modeling for Single-Cell Transcriptomics.” Nature Methods 15 (12): 1053–58.*

*Luecken, Malte D., and Fabian J. Theis. 2019. “Current Best Practices in Single-Cell RNA-Seq Analysis: A Tutorial.” Molecular Systems Biology 15 (6): e8746.*

*Stein-O’Brien, Genevieve L., Brian S. Clark, Thomas Sherman, Cristina Zibetti, Qiwen Hu, Rachel Sealfon, Sheng Liu, et al. 2019. “Decomposing Cell Identity for Transfer Learning across Cellular Measurements, Platforms, Tissues, and Species.” Cell Systems 8 (5): 395–411.e8.*

*Tabula Muris Consortium. 2020. “A Single-Cell Transcriptomic Atlas Characterizes Ageing Tissues in the Mouse.” Nature 583 (7817): 590–95.*

**Second round of review**

**Reviewer 1**

I would like to thank the authors for addressing all my comments. I have only minors remark with respect to the cell ontologies. I commend the authors on the adopted solution to rely on standardized ontologies as well as providing a simple label translation tool.

- Perhaps it is useful to add a small remark regarding the cell types covered by The Cell Ontology. Despite being the most extensive ontology with more than 2000 cell types, for certain tissues (e.g. brain) it still lags significantly behind the deep level of annotation used in single-cell studies.

- And regarding the label translation tool, perhaps future work can use topological relations in the ontology to improve mapping accuracy. Textual labels suffer from many inconsistencies and as it stands, sfaira will be dependent on the quality of the originally assigned labels which can be misleading in many cases. Examples of work that rely on the Ontology topology include OnClass (https://doi.org/10.1101/810234) and CellO (https://doi.org/10.1016/j.isci.2020.101913).

**Reviewer 2**

The newly added analyses/changes and the description on the methodological details have greatly improved the clarity of the work. The updated software documentation has improved the reproducibility.
